# Arylsulphatase A Pseudodeficiency (ARSA-PD), hypertension and chronic renal disease in Aboriginal Australians

**DOI:** 10.1038/s41598-018-29279-9

**Published:** 2018-07-19

**Authors:** Dave Tang, Michaela Fakiola, Genevieve Syn, Denise Anderson, Heather J. Cordell, Elizabeth S. H. Scaman, Elizabeth Davis, Simon J. Miles, Toby McLeay, Sarra E. Jamieson, Timo Lassmann, Jenefer M. Blackwell

**Affiliations:** 1 Telethon Kids Institute, The University of Western Australia, Subiaco, Western Australia 6008 Australia; 2 National Institute of Molecular Genetics, Milan, Italy; 3 Institute of Genetic Medicine, Newcastle University, Newcastle upon Tyne, NE1 3BZ United Kingdom; 4 Department of Endocrinology and Diabetes, Princess Margaret Hospital for Children, Subiaco, Western Australia 6008 Australia; 5 Ngangganawili Aboriginal Health Service, Wiluna, Western Australia 6646 Australia

## Abstract

Chronic renal disease (CRD) associated with cardiovascular disease (CVD) and/or type 2 diabetes (T2D) is a significant problem in Aboriginal Australians. Whole exome sequencing data (N = 72) showed enrichment for ClinVar pathogenic variants in gene sets/pathways linking lipoprotein, lipid and glucose metabolism. The top Ingenuity Pathway Analysis canonical pathways were Farsenoid X Receptor and Retinoid Receptor (FXR/RXR; (P = 1.86 × 10^−7^), Liver X Receptor and Retinoid Receptor (LXR/RXR; P = 2.88 × 10^−6^), and atherosclerosis signalling (P = 3.80 × 10^−6^). Top pathways/processes identified using Enrichr included: Reactome 2016 chylomicron-mediated lipid transport (P = 3.55 × 10^−7^); Wiki 2016 statin (P = 8.29 × 10^−8^); GO Biological Processes 2017 chylomicron remodelling (P = 1.92 × 10^−8^). ClinVar arylsulfatase A pseudodeficiency (ARSA-PD) pathogenic variants were common, including the missense variant c.511 G > A (p.Asp171Asn; rs74315466; frequency 0.44) only reported in Polynesians. This variant is in *cis* with known ARSA-PD 3′ regulatory c.*96 A > G (rs6151429; frequency 0.47) and missense c.1055 A > G (p.Asn352Ser; rs2071421; frequency 0.47) variants. These latter two variants are associated with T2D (risk haplotype GG; odds ratio 2.67; 95% CI 2.32–3.08; *P* = 2.43 × 10^−4^) in genome-wide association data (N = 402), but are more strongly associated with quantitative traits (DBP, SBP, ACR, eGFR) for hypertension and renal function in non-diabetic than diabetic subgroups. Traits associated with CVD, CRD and T2D in Aboriginal Australians provide novel insight into function of ARSA-PD variants.

## Introduction

Chronic (CRD) and end stage (ESRD) renal disease associated with cardiovascular disease (CVD) and/or type 2 diabetes (T2D) are major health problems in Aboriginal Australians^1,2^. Around 10% of new cases of ESRD are Aboriginal Australians, despite making up only 2.5% of the population^3^. Aboriginal Australians present with ESRD at a younger age, with 70% of Indigenous cases occurring at <60 years compared to 20% for non-Indigenous Australians^3^. The most common causes of ESRD are T2D, glomerulonephritis, and hypertension (HTN)^3^. A body mass index (BMI) > 22 kg/m^2^ is a significant risk factor for T2D in Aboriginal Australians^2^. BMI is also a risk factor for ESRD independently of diabetes^4^.

Genome-wide association studies (GWAS) have been used successfully in large-scale cohorts to identify common genetic variants associated with BMI and T2D^5^, and for quantitative traits (QT) related to CVD/HTN or CRD/ESRD^6,7^. We undertook the first GWAS looking for common variants associated with BMI and T2D in Aboriginal Australians^8^, and found evidence for genes/pathways in common with genetic risk factors in other ethnicities. This included top hits for BMI 5′ of *NTRK2*, the type 2 neurotrophic tyrosine kinase receptor for brain-derived neurotrophic factor that regulates energy balance downstream of melanocortin-4 receptor, and for T2D 5′ of *BCL9* that, along with *TCFL2*, promotes beta-catenin’s transcriptional activity in the WNT signalling pathway. However, our study was not well-powered to provide definitive support for other hits that occurred genome-wide, and we did not look at possible roles of known functional variants, including rare variants, that may contribute to genetic risk for T2D, HTN/CVD or CRD/ESRD. Cumulative evidence highlights the contribution of less common or rare functional variants to complex diseases^9–12^, particularly in family-based analyses^11,13^. ClinVar is a public archive that catalogues the relationships between putative functional variants and human disease phenotypes^14^. Here we use whole exome sequencing (WES) to identify putative functional variants that may contribute to genetic risk for CRD/ESRD associated with CVD/HTN and/or T2D in this Australian Aboriginal population. We find enrichment for genes carrying ClinVar functional variants in gene sets/pathways that link bile acid regulation with lipoprotein, lipid and glucose metabolism. In addition, functional variants causing arylsulfatase A pseudodeficiency (ARSA-PD) are common in this population, are associated with T2D, and more strongly with QT for CVD/HTN and CRD/ESRD in non-diabetic individuals.

## Results

### Identifying functional variants using WES

Our initial focus was to analyse WES data from the 72 individuals (see methods) to identify putative functional variants *per se* that would provide an important population-specific resource for clinical diagnosis of rare disease variants in the Aboriginal population^15,16^. Firstly, we compared all variants called in our WES data with functional disease-associated variants in ClinVar^14^. Supplementary Table S1 shows all ClinVar variants designated as pathogenic, likely pathogenic, or a risk factor in at least one study, with variant frequencies compared to Max-all. These variants ranged from deleterious variants of high burden that have previously been recorded as pathogenic for rare genetic disorders, to those contributing to quantitative and/or more complex phenotypes such as the cardiovascular and renal disease phenotypes of specific interest to this study (cf. below). Of interest, individuals were observed (summarised Table 1; presented in full in Supplementary Table S1) who were heterozygous for putative high burden genetic variants/disorders previously identified as autosomal dominant, while individuals homozygous for high burden variants were also observed for genetic disorders previously reported as autosomal recessive. Some of these may be of lower burden and could represent undiagnosed cases, while others may already be associated with conflicting interpretations of pathogenicity recorded in ClinVar. However, since none of the participants in the study presented with severe rare genetic disorders, the observed genotype frequencies for variants in this Aboriginal Australian population may also call into question the pathogenicity status of some that are recorded as high burden pathogenic variants in ClinVar. Clinically accredited sequence analysis will be required to determine the potential impact of these variants in Aboriginal Australians presenting with rare genetic disorders. The de-identified research sequence data obtained here has been made available (see methods for details of data access) as a unique population frequency baseline for clinical diagnosis of rare disease variants in the Aboriginal population^15,16^.

**Table 1:** Summary of genotype frequencies in the Aboriginal Australian study population for putative high burden pathogenic variants.

Chrom	Gene	SNP ID	cDNA	Protein	Genotype Freq			MoI	Clinvar disease name/Phenotype
					Hom Ref	Het	Hom Var		
**Dominant disorders:**									
chr3	SCN5A	rs199473118 rs1805124	c.1535 C > T c.1673A > G	p.Thr512Ile p.His558Arg	54	15	3	AD	Progressive familial heart block type 1 A (haplotype)
chr4	DSPP	rs36094464	c.202 A > T	p.Arg68Trp	67	5	0	AD	Dentinogenesis imperfecta-Shield’s type II
chr6	HFE	rs1800562	c.845 G > A	p.Cys282Tyr	66	6	0	AD	Porphyria cutanea tarda; Porphyria variegata
chr9	COL5A1	rs61735045	c.1588 G > A	p.Gly530Ser	65	7	0	AD	Ehlers-Danlos syndrome, classic_type
chr10	LDB3	rs145983824	c.1823C > T	p.Pro608Leu	65	7	0	AD	Familial hypertrophic cardiomyopathy 24
chr10	ZFYVE27	rs35077384	c.572 G > T	p.Gly191Val	59	13	0	AD	Spastic paraplegia 33, autosomal dominant
chr11	TYR	rs1126809	c.1205 G > A	p.Arg402Gln	63	9	0	CH; DG	Oculocutaneous albinism 1/1B (CH); Waardenburg syndrome 2 and ocular albinism, digenic
chr11	ROM1	rs527236104	c.331dupG	p.Leu114Alafs	63	8	1	DG	Retinitis pigmentosa 7, digenic with RDS
chrX	OFD1	rs398122866	c.688_705del18	p.Ile230_Lys235del	41	2	0	XLD	Oral-facial-digital syndrome
**Recessive disorders:**									
chr1	FMO3	rs2266782	c.472 G > A	p.Glu158Lys	14	23	35	AR	Trimethylaminuria
chr1	GNPAT	rs11558492	c.1556 A > G	p.Asp519Gly	39	27	6	AR	Rhizomelic chondrodysplasia punctata 2
chr9	ADAMTS13	rs2301612	c.1342 C > G	p.Gln448Glu	16	37	19	AR	Upshaw-Schulman syndrome
chr4	KLKB1	rs3733402	c.428 G > A	p.Ser143Asn	32	29	11	AR	Prekallikrein deficiency
chr7	CFTR	rs727504486	c.1210-12_1210-6T [5]	NA	26	29	8	AR	Absence vas deferens; Cystic_fibrosis
chr18	FECH	rs2272783	c.315-48 T > C	NA	42	26	4	AR	Erythropoietic protoporphyria; Erythema
chr17	GAA	rs1800309	c.2065 G > A	p.Glu689Lys	46	21	5	AR	Acid alpha-glucosidase, allele 4
chr5	IL7R	rs1494558	c.197 T > C	p.Ile66Thr	23	35	14	AR	Severe combined immunodeficiency
chr5	RARS	rs139644798	c.1367 C > T	p.Ser456Leu	62	9	1	AR	Leukodystrophy, hypomyelinating, 9
chr6	ESR1	rs6929137	c.1810G > A	p.Val604Ile	33	30	9	AR	Estrogen resistance
chr7	ATP6V0A4	rs3807153	c.1739T > C	p.Met580Thr	40	26	6	AR	Renal tubular acidosis, distal
chr12	ACADS	rs1799958	c.625 G > A	p.Gly209Ser	21	38	13	AR	Deficiency of butyryl-CoA dehydrogenase
chr14	RPGRIP1	rs10151259	c.1639G > T	p.Ala547Ser	24	32	16	AR	Cone-rod dystrophy 13
Chr19	GCDH	rs8012	c.1250 A > G	p.Gln417Arg	9	30	33	AR	Glutaric aciduria, type_1
chr19	GCDH	rs9384	c.*288 G > T	NA	14	28	30	AR	Glutaric aciduria, type_1
chr19	MAG	rs2301600	c.399 C > G	p.Ser133Arg	49	22	1	AR	Spastic paraplegia 75

Complete data are provided in Supplementary Table S1. Summary data are provided here for variants in genes for which severe genetic disorders with autosomal (AD) or X-linked (XLD) dominant (where ≥4 heterozygotes were observed) or autosomal recessive (where ≥1 homozygote was observed) inheritance are reported (OMIM). Note for the one XLD variant at OFD1 the genotype frequencies given are for females; there were 27 males carrying the wildtype allele. Chrom = chromosome; SNP ID - rs ID as in DbSNP; cDNA = change caused by variant at cDNA level; Protein = change caused by variant at protein level; Genotype Freq = genotype frequencies for homozyzygous reference allele, heterozygotes, and homozygous variant allele; MoI = mode of inheritance (AD = autosomal dominant; XLD = X-linked dominant; AR = autosomal recessive; CH = compound heterozygotes; DG = digenic); ClinVar disease name = disease for which ClinVar records the pathogenicity assignment indicated.

Secondly, since there may be novel deleterious variants in the Australian Aboriginal population not present, or at very low frequency, in ClinVar or other public domain databases, we assigned CADD^17^ values to all variants called in the WES data and filtered for genes carrying potential deleterious variants. The top 5% of putative deleterious variants had scaled CADD scores ≥ 15.91, which aligns with the median value for all possible canonical splice site changes and non-synonymous variants in the human genome that has been suggested as a potential cut-off for pathogenicity in population-based studies^17^. The top 10% had scaled CADD scores ≥12.97. Those present (N = 352) at variant allele frequencies >0.2 in the study population, but with Max-all <0.1, are presented in Supplementary Table S2. Of these, 82 (listed as Vaf = −1) were not found in the databases used to determine Max-all, and 27 (listed as Vaf = 0) had Max-all <0.001.

### Enrichment for ClinVar functional variants regulating lipoprotein, lipid and glucose metabolism

**Figure 1:**
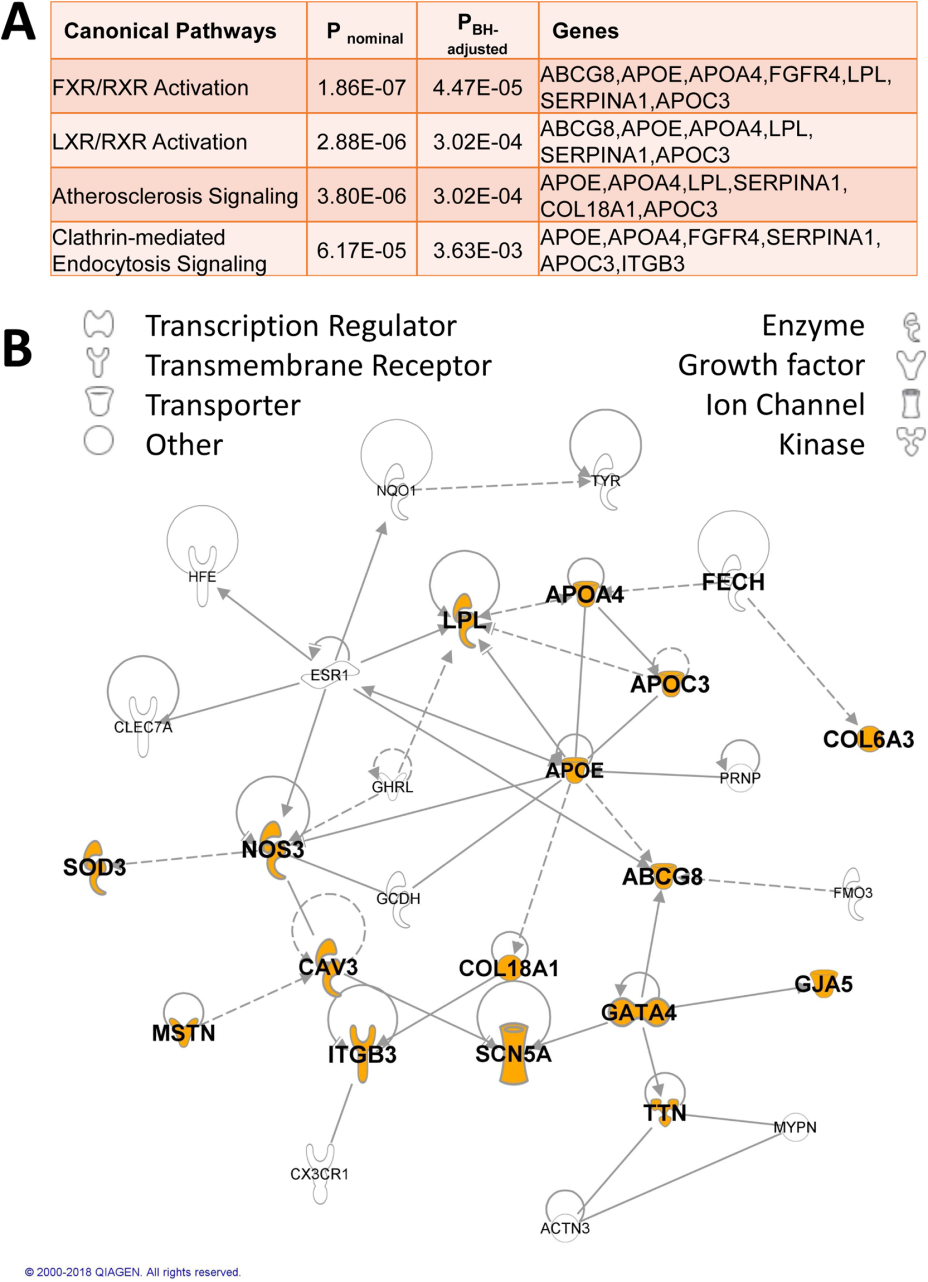
Results of canonical pathway and network analyses for 111 genes carrying 125 ClinVar functional variants. (**A**) Top canonical pathways analysed using QIAGEN’s Ingenuity Pathway Analysis (IPA; QIAGEN Inc., https://www.qiagenbioinformatics.com/products/ingenuity-pathway-analysis)^52^ are shown along with nominal and adjusted p-values, and genes within our dataset involved in the listed pathways. (**B**) Plot showing the network of interconnected genes identified by Network Analysis undertaken using IPA. Genes with no previously documented interactions were removed from the diagram. The network contains 29 (40%) of the genes carrying ClinVar variants selected for pathway analysis, 16 of which (annotated in orange) are members of the top canonical pathways listed in (**A**) and/or enriched gene sets (Supplementary Table S3) involved in functions related to CVD.

Using Enrichr^18^ (Supplementary Table S3) and IPA (Fig. 1) we looked for enrichment of gene sets and pathways in 72 genes containing 81 ClinVar pathogenic variants of lower burden than those definitively known to cause severe congenital genetic disorders (e.g. as discussed above in relation to Table 1). In addition to a general enrichment for genes involved in human metabolism as compared to the Reactome 2016 (adjusted P = 1.25 × 10^−6^) and KEGG 2016 (adjusted P = 1.43 × 10^−4^) databases, there was consistent evidence across all databases for enrichment of genes involved in lipid, lipoprotein and retinoid metabolism and transport (Supplementary Table S3). This was also concordant with the top canonical pathways for Farsenoid X Receptor and Retinoid Receptor (FXR/RXR), Liver X Receptor and Retinoid Receptor (LXR/RXR), and atherosclerosis signalling, identified using IPA (Fig. 1A). Network analysis in IPA (Fig. 1B) summarises how these genes interact. There was no significant enrichment for gene sets or pathways for the 136 genes carrying the top 5% of variants with scaled CADD score ≥ 15.91 and variant allele frequencies >0.2 in our study and Max-all <0.1. It is of note, nevertheless, that not all variants identified as pathogenic in ClinVar are robust to this stringent scaled CADD score cut-off (Supplementary Table S1). Lowering the threshold to look at the 301 genes carrying the top 10% of variants with scaled CADD score ≥ 12.97 and with variant allele frequencies >0.2 in our study and Max-all <0.1 (Supplementary Table S2) showed significant enrichment for genes (*DPH3, DRD2, LIF, OSM, RHBDF2*; variant allele frequencies 0.236 to 0.806; Max-all <0.088; CADD-scaled 13.08 to 22.3) in the GO Biological Process 1917b database for negative regulation of adiponectin secretion (Enrichr nominal P = 5.4 × 10^−6^, adjusted P = 0.019). No other gene sets were robust to adjusted P < 0.05 in Enrichr or in IPA canonical pathway analysis (data not shown).

### ARSA-PD variants in the study population

Amongst the pathogenic ClinVar variants present at high frequency in this Australian Aboriginal population was the ARSA missense variant c.511 G > A (p.Asp171Asn; rs74315466; causal allele frequency 0.44; Supplementary Table S1) reported as pathogenic for ARSA-PD in ClinVar. This variant (present in 1000 G at frequency 0.0049 in Indian Teluga from UK, and at 0.0156 in Punjabi from Lahore) had only been previously reported^19^ as an unpublished observation in a Polynesian population in *cis* with a well-known ARSA-PD 3′ regulatory variant c.*96 A > G (rs6151429) which is also present at a high frequency (0.47; Table 2) in our population compared to other ethnicities. These two variants are in strong linkage disequilibrium (LD; r^2^ = 0.88) with each other in our WES data. A second missense variant c.1055 A > G (p.Asn352Ser; rs2071421; traditionally named Asn350Ser) also occurred in *cis* with the PD-allele at rs6151429 (r^2^ = 1) in our WES data, consistent with the hypothesis that this 3′ polyadenylation regulatory variant c.*96 A > G arose on the background of the more ancient c.1055 A > G variant before the emergence of modern *Homo sapiens* from Africa^20^. Table 2 provides allele frequencies for the 2 well known ARSA-PD variants, rs6151429 and rs2071421, in our study compared against different ethnicities in the public domain databases.

**Table 2:** Population frequencies for ARSA-PD variants rs6151429 and rs2071421.

SNP	Population	Variant Allele Count	2 N Total Allele Count	N Homozygotes for variant allele	Variant Allele Frequency
rs6151429; c.*96 A > G	Aboriginal Australia WD	68	144	14	0.472
	AFR	5	1322	0	0.004
	AMR	28	694	0	0.040
	EAS	18	1008	1	0.018
	EUR	82	1006	3	0.082
	SAS	117	978	7	0.120
rs2071421; c.1055 A > G p.Asn352Ser	Aboriginal Australia WD	68	144	14	0.472
	AFR	499	1322	104	0.377
	AMR	175	694	31	0.252
	EAS	161	1008	9	0.160
	EUR	140	1006	12	0.140
	SAS	151	978	11	0.154

Frequencies for Australian Aboriginals (WD = Western Desert, this study) are compared to different ethnicities in the 1000 Genomes project, including African/African American (AFR), Latino (AMR), East Asian (EAS), European (EUR), and South Asian (SAS).

The absence of the ARSA missense variant c.511 G > A (p.Asp171Asn; rs74315466) from ExAC, which contains multiple ethnicities including African/African American (AFR), Latino (AMR), East Asian (EAS), Finnish (FIN), Non-Finnish European (NFE), and South Asian (SAS), suggests that this variant may be unique to Aboriginal Australians and Polynesian/Indian ethnicities, where it has arisen on the background of the classical ARSA-PD alleles of both the 3′ regulatory c.*96 A > G (rs6151429) and the c.1055 A > G (p.Asn352Ser; rs2071421) variants. This is also consistent with data from the first whole genome sequence of an Aboriginal Australian^21^ where the individual sequenced was homozygous for the PD-associated variant alleles at all 3 SNPs (rs6151429, rs2071421, rs74315466; data available at http://www.cbs.dtu.dk/public/aboriginal/genotyping/AusAboriginal/?C=S;O=A).

### ARSA-PD variants associated with T2D

Arylsulfatase A (cerebroside-3-sulfate 3-sulfohydrolase; EC 3.1.6.8) is a lysosomal enzyme that plays a role in catalysing the degradation of sulfatides, a subgroup of glycosphingolipids important in diabetes^22^. Since both rs6151429 and rs2071421 were present on the Illumina 2.5 M Duo beadchip used in our GWAS of 402 post-QC individuals^8^, we re-interrogated the data to determine whether these ARSA-PD variants were associated with T2D in this Aboriginal population. In this larger dataset, these two variants were in complete LD (r^2^ = 1) in 78 unrelated individuals of pure Martu ancestry, and in very strong LD (r^2^ = 0.99) in 146 unrelated individuals in this Aboriginal/Caucasian admixed population^8^. Linear Mixed Model analysis of 89 T2D cases ≥ 20 years old in the family-based dataset of 391 GWAS individuals^8^ showed association with both rs2071421 (P = 5.49 × 10^−4^) and rs6151429 (P = 8.90 × 10^−4^) (data not shown). Logistic regression analysis of these 89 cases compared to 109 unaffected adults (≥20 years old) (Supplementary Table 3A) under an additive model concurred with data from the original GWAS, with the top associations at rs2071421 (odds ratio risk G allele = 2.73; 95% confidence interval 1.62–4.60; P = 1.54 × 10^−4^), rs6151429 (odds ratio risk G allele = 2.67; 95% confidence interval 1.58–4.52; P = 2.43 × 10^−4^) and other SNPs in complete LD with rs6151429. Analysis under dominant (rs2071421 odds ratio risk G allele = 3.41; 95% confidence interval 1.65–7.04; P = 8.96 × 10^−4^) or recessive (rs2071421 odds ratio risk G allele = 3.421; 95% confidence interval 1.37–8.53.60; P = 8.23 × 10^−3^) models provided higher odds ratios but larger variance and reduced significance. Association with T2D under an additive model is consistent with previous work demonstrating that individuals heterozygous for ARSA-PD variants have intermediate ARSA enzyme activity^23^.

Haplotype association analysis (Supplementary Table S4) of phased haplotypes across the 6 *ARSA* variants at the peak of the association demonstrated that T2D risk is associated with the haplotype AAGGAA (odds ratio 2.67; P = 3.21 × 10^−4^) that carries G alleles at rs2071421 and rs6151429 previously associated with ARSA PD. Protection was associated with the haplotype GGAAGG (odds ratio 0.468; P = 2.77 × 10^−3^). In a sliding window analysis of pairwise haplotypes across the 6 SNPs (Supplementary Table S4) the peak risk haplotype (GA; odds ratio 2.91; P = 7.61 × 10^−5^) was observed across SNPs rs2071421 and rs6151419, raising the possibility that the more 5′ *ARSA* missense variant c.511 G > A (p.Asp171Asn; rs74315466) that lies distal to rs6151419 could contribute to the association. However, while conditioning on rs2071421 or rs6151429 removes significance at all other SNPs, conditioning on rs6151419 retains residual signals at both rs2071421 and rs6151429 (Table 3B). Hence, it seems likely that the association is fully accounted for by the two known ARSA-PD variants rs2071421 and rs6151429.

**Table 3:** Logistic regression analysis of association between ARSA SNPs and T2D.

SNP	BP	A1	OR	SE	L95	U95	STAT	P
**(A)**								
rs11912237	51060049	A	2.67	0.27	1.58	4.52	3.67	2.43E-04
rs8142033	51062832	A	2.67	0.27	1.58	4.52	3.67	2.43E-04
rs6151429	51063477	G	2.67	0.27	1.58	4.52	3.67	2.43E-04
rs2071421	51064416	G	2.73	0.27	1.62	4.60	3.79	1.54E-04
rs6151419	51064915	A	2.23	0.26	1.35	3.68	3.14	1.72E-03
rs762668	51066990	A	2.52	0.27	1.49	4.27	3.44	5.92E-04
**(B)**								
	**BP**	**Original P**	**P after conditioning on**					
			**rs6151429**		**rs2071421**		**rs6151419**	
rs6151429	51063477	2.43E-04	1		1		0.05	
rs2071421	51064416	1.54E-04	1		1		0.03	
rs6151419	51064915	1.72E-03	0.96		0.74		1	
rs762668	51066990	5.92E-04	0.84		0.80		0.10	

Analysis performed in PLINK using an additive model with 10 principal components of variation from the original GWAS analysis^8^ used as covariates. Summary data for the top SNP under dominant and recessive models is provided in the text. SNP = variant analysed; BP = bp location Build 36; A1 is associated allele; OR = odds ratio; SE = standard error; L95 and U95 = lower and upper 95% confidence intervals; STAT = test statistic; P = P-values. (A) are results for single SNP association analyses; (B) are results after conditioning on each SNP, as indicated.

### ARSA-PD variants associated with HTN- and CRD-related quantitative traits

**Figure 2:**
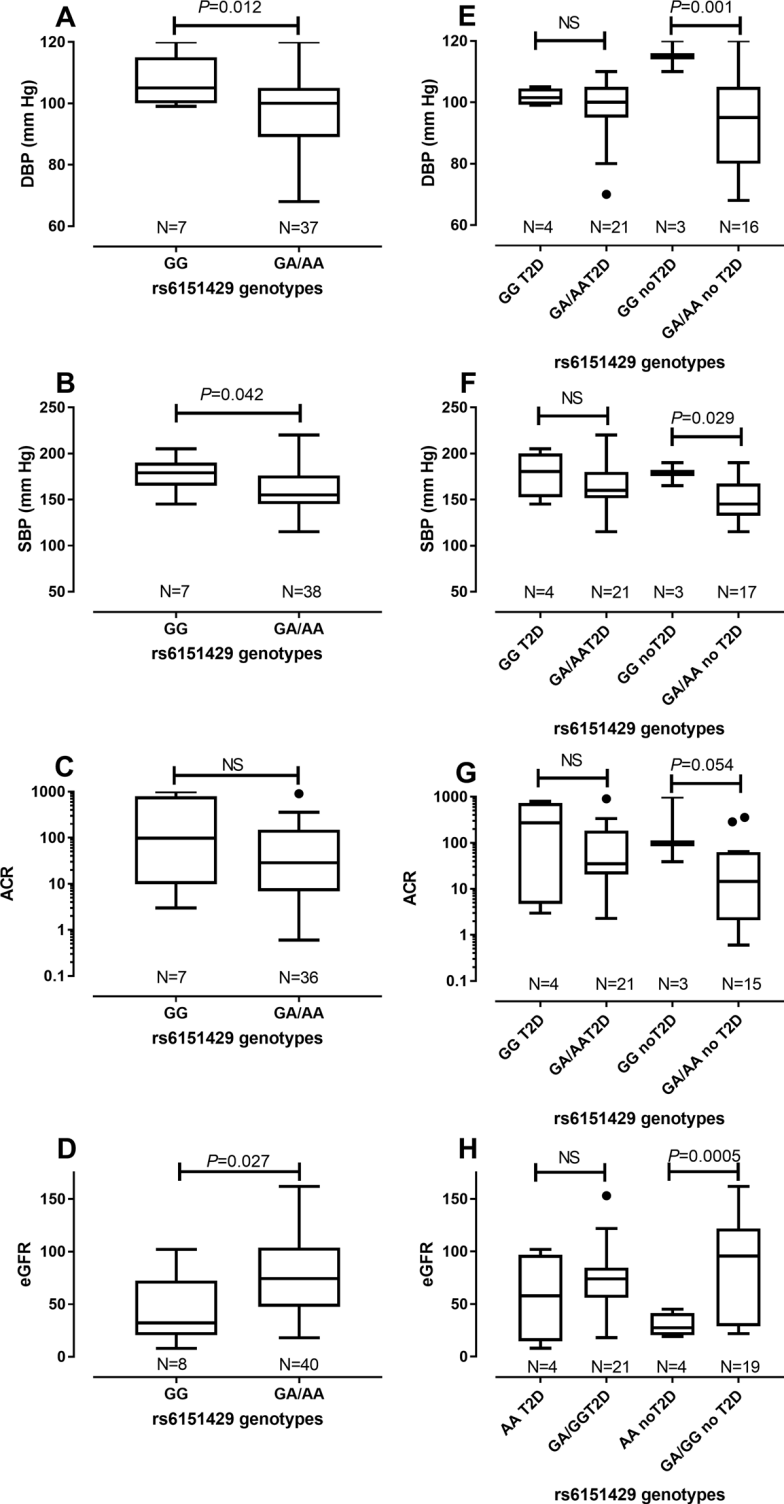
Relationship between ARSA rs6151429 genotypes and QT measures of HTN (DBP and SBP) or renal function (ACR and eGFR) in the study population. (**A**) to (**D**) show Box and Whiskers Tukey plots for genotype by QTs for adult WES participants; (**E**) to (**H**) show results for adult WES participants stratified by T2D status.

Serum sulfatides are also a biomarker for CVD, particularly in the context of ESRD^24^. We therefore looked for associations between ARSA-PD genotypes and quantitative traits (QT) associated with HTN (DBP; SBP) and renal function (ACR; eGFR). We also looked at associations with HbA1c used to monitor diabetes risk, and BMI as a risk factor for T2D. Since the ARSA-PD 3′ regulatory variant c.*96 A > G (rs6151429) has been shown experimentally to be the etiological variant for low ARSA activity associated with pseudodeficiency^25^, and the three variants rs6151429, rs2071421 and rs74315466 are in almost complete LD in our population, all associations with QT data are presented relative to rs6151429 genotypes. The 48 adults analysed by WES were selected for extremes of eGFR with/without T2D (Supplementary Table S5). We therefore looked initially for associations between rs6151429 genotypes and QT in this sample. DBP (Mean difference −10.7; 95% CI −18.56 to −2.836; P = 0.012), SBP (Mean difference −19.33; 95% CI −19.33 to −0.88; P = 0.042) and eGFR (Mean difference 32.99; 95% CI 4.55 to 61.44; P = 0.027) were significantly associated with rs6151429 genotypes (Fig. 2). In each case the direction of differences is as expected, with the ARSA-PD GG genotype (i.e. homozygous for PD deficiency) associated with at risk higher DBP and SBP, and lower eGFR. ACR was not significantly associated with rs6151429 genotypes in the total WES adult (N = 48) sample. Of note, associations between rs6151429 genotypes and DBP (Mean difference −20.75; 95% CI −31.28 to −10.22; P = 0.001), SBP (Mean difference −27.88; 95% CI −51.57 to −4.19; P = 0.029), ACR (Median difference −83.4; P = 0.054) and eGFR (Mean difference 49.95; 95% CI 24.83 to 75.07; P = 0.0005) were evident in the non-diabetic group but not the group with T2D (Fig. 2). Notably, HbA1c (Supplementary Fig. S2) and BMI (Supplementary Fig. S3) were not associated with rs6151429 genotypes, with or without stratification for T2D. These data suggest that the ARSA PD allele is acting directly on CVD and CRD, rather than as an indirect effect of its association with T2D as observed in the GWAS data. Conversely, the association with T2D in the GWAS dataset may be due to correlation with CVD- and CRD-related traits.

**Figure 3:**
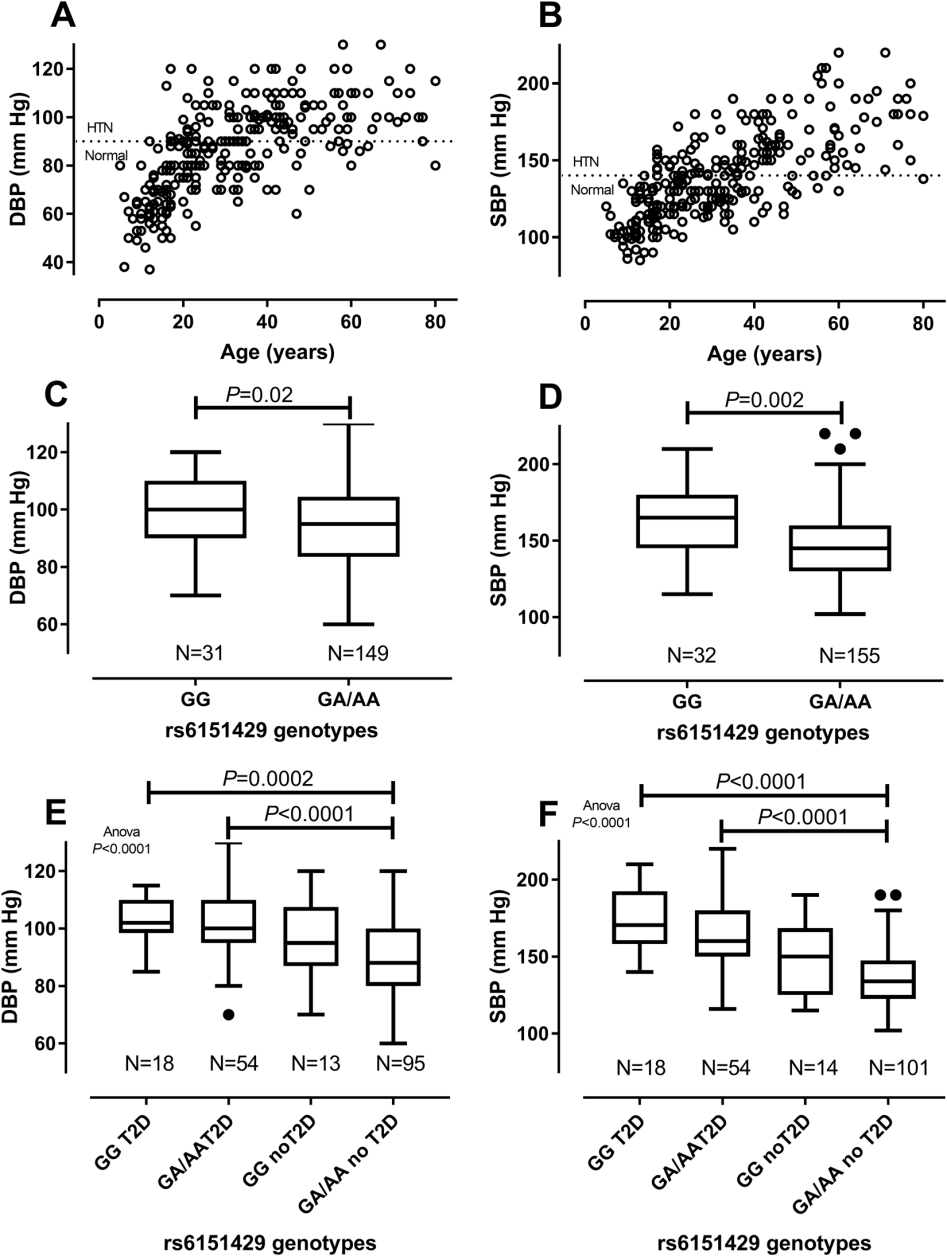
Relationship between ARSA rs6151429 genotypes and QT (DBP and SBP) measures of HTN in the GWAS population. (**A**) and (**B**) show DBP and SBP by age for all individuals contributing to the GWAS; dotted lines indicate clinical cut-offs for HTN. (**C**) and (**D**) show Box and Whiskers Tukey plots for genotype by DBP and SBP, respectively; (**E**) and (**F**) show results stratified by T2D status.

**Figure 4:**
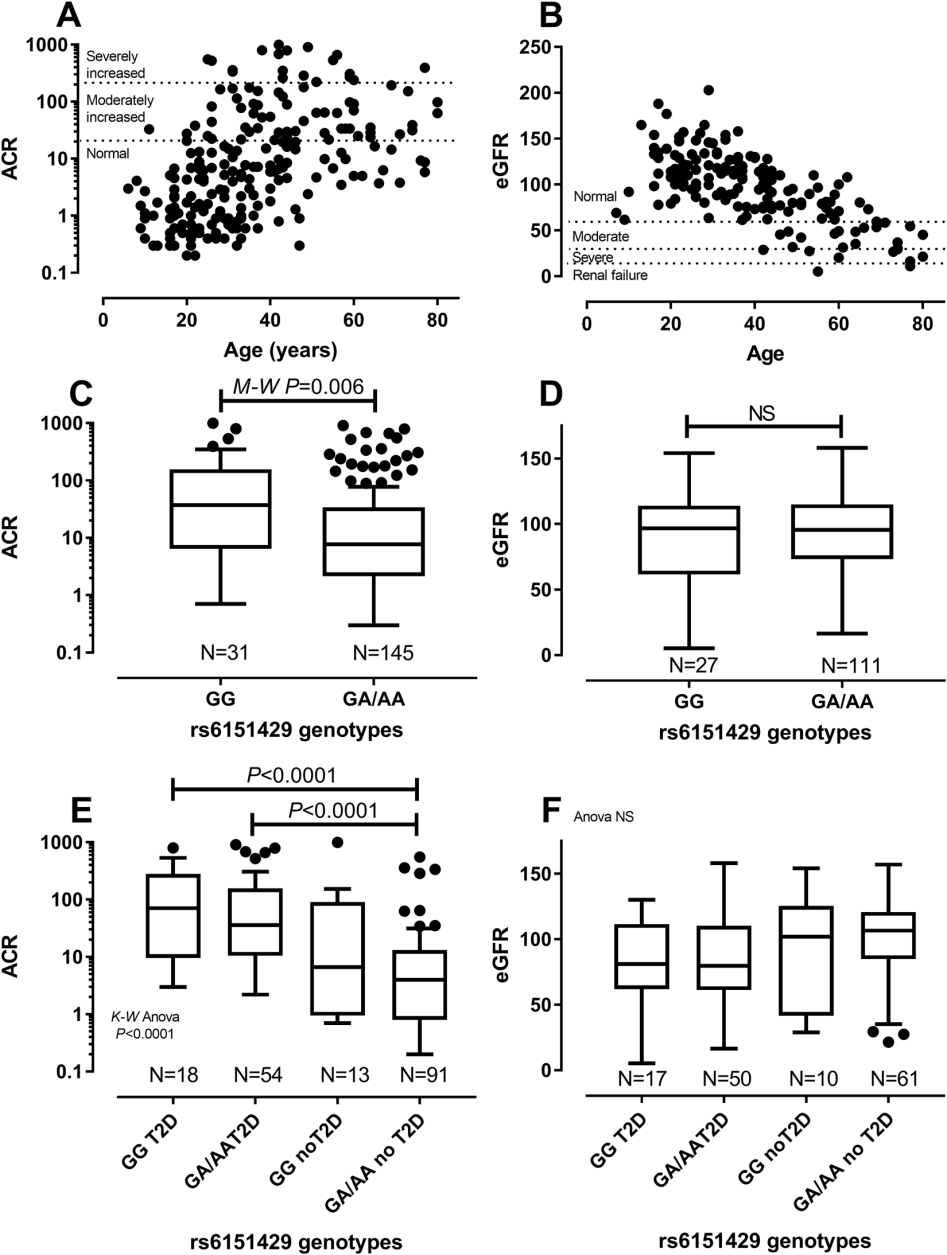
Relationship between ARSA rs6151429 genotypes and QT (ACR and eGFR) for renal function in the GWAS population. (**A**) and (**B**) show ACR and eGFR by age for all individuals contributing to the GWAS; dotted lines indicate clinical cut-offs for renal function as annotated. (**C**) and (**D**) show Box and Whiskers Tukey plots for genotype by ACR and eGFR, respectively; (**E**) and (**F**) show results stratified by T2D status.

Since rs6151429 was genotyped in all individuals used in the GWAS, we also examined associations with HTN (DBP, SBP) and CRD (ACR, eGFR) QT, as well as BMI and HbA1c, in this larger data set. Figure 3A,B showing DBP and SBP by age indicate that values only exceed clinical thresholds for HTN in adulthood. Associations between ARSA-PD genotypes and QT were therefore restricted to individuals aged ≥ 20 years. Significant associations between rs6151429 genotype and DBP (Mean difference −5.63; 95% CI −10.34 to −0.92; P = 0.02) or SBP (Mean difference −16.6; 95% CI −26.49 to −6.72; P = 0.002) observed in the full dataset (Fig. 3C,D) were not evident following stratification by T2D (Fig. 3E,F). Maximum between group differences were observed when each T2D group was compared with the non-T2D group carrying the protective non-ARSA-PD allele, suggesting that T2D disease was neutralising any protection afforded by genotypes heterozygous or homozygous for the non-PD allele. As for DBP and SBP, ACR and eGFR only exceed clinical thresholds for CRD in adulthood (Fig. 4A,B). The CRD-related trait ACR was significantly associated with rs6151429 genotype in the total group (Fig. 4C). Significance between genotypes and ACR were not evident once stratified by T2D (Fig. 4E), with maximum differences in ACR levels again observed when comparing the two T2D groups with the non-T2D group carrying the protective non-ARSA-PD allele. No significant associations between eGFR and rs6151429 genotype were observed in this larger dataset (Fig. 4D,F), nor with HbA1c (Supplementary Fig. S1) or BMI (Supplementary Fig. S2). Absence of evidence for any direct association between rs6151429 genotype and HbA1c lends weight to the suggestion that the functional association related to CVD/HTN and CRD is direct, and not indirectly through association with T2D.

## Discussion

Few studies have used genome-wide analyses of Australian Aboriginal-ancestry populations to identify variants contributing to high rates of CVD, CRD/ESRD and T2D. Amongst such variants we expect to observe some that are Australian Aboriginal-specific, or at higher frequency in this population, as has been observed, for example, for kidney disease^26^ and blood pressure^27^ in African-ancestry populations. In a family-based study, it was also likely that multiple rare variants might contribute to these phenotypes, as recently observed for blood pressure in families from the Cleveland family Study^13^. Using WES analysis of family members with extreme phenotypes we showed enrichment for genes carrying ClinVar functional variants in gene sets/pathways that link bile acid regulation with lipoprotein, lipid and glucose metabolism. Notable amongst ClinVar functional variants contributing to enriched gene sets/pathways, and occurring at high frequency, was APOE rs429358 (c.388 T > C; p.Cys130Arg; frequency 0.326; also referred to as ApoE4, epsilon 4 or Cys112Arg) which is associated with familial_type_3_hyperlipoproteinemia^28^. The Max-all frequency of 0.273 in our database comparison (Table S2 in the Supplementary Data) is for African ancestry, while European frequencies are ∼0.17 and Asian ∼0.09. APOE variants are associated with autosomal dominant and multifactorial inheritance of type 3 hyperlipoproteinemia. Understanding this genetic disease has been key to identifying the role of APOE in removal very low density lipoprotein and chylomicrons^28^. ApoE4 is associated with atherosclerosis^29^, Alzheimer’s disease^30^ and impaired cognitive performance^31^. A second ClinVar functional variant at high frequency in our study was *ATP6V0A4* rs3807153 (c.1739T > C; p.Met580Thr; frequency 0.264) which is associated with distal renal tubular acidosis^32^. Again, the high Max-all frequency (0.213; Table S2 in the Supplementary Data) is representative of African ethnicities, while Europeans (0.01–0.02) and Asians (0.05–0.06) have low frequencies. Using CADD scaled scores to identify putative functional variants unique to, or at high frequency, in this Australian Aboriginal population showed enrichment for genes (*DPH3, DRD2, LIF, OSM, RHBDF2*) contributing to negative regulation of adiponectin secretion. Adiponectin is an adipocyte-derived plasma protein with insulin-sensitizing, anti-atherosclerotic and anti-diabetic properties^33,34^. Adiponectin secretion from omental cells is high, sensitive to insulin, and negatively correlated with BMI^33^. Serum adiponectin is higher in nondiabetics with renal insufficiency than without^34^, and has been proposed as a biomarker of CVD^35^. Larger well-powered studies are required to determine the broader impact of these CADD and ClinVar functional variants on CVD and CRD/ESRD in Aboriginal Australians.

We noted that ARSA-PD variants are common in this Australian Aboriginal population. Numerous deleterious protein coding *ARSA* gene variants are associated with metachromatic leukodystrophy (MLD), a rare lysosomal storage disorder^36^. Although ARSA-PD variants are not associated with MLD *per se*, PD variants occurring in *cis* with MLD-causing mutations could exacerbate the MLD phenotype^37^. Nevertheless, the 3′ regulatory c.*96 A > G (rs6151429) ARSA-PD variant, which severely reduces a 2.1-kilobase mRNA species to give ARSA enzyme levels ∼10% of normal, is commonly observed in healthy individuals^25^. The associated c.1055 A > G (p. Asn352Ser; rs2071421; traditionally known as p.Asn350Ser) variant causes the loss of an N-linked glycosylation site, affecting transport to the lysosome. Although c.1055 A > G does not itself reduce enzyme activity^38^, the combined effect of the polyadenylation defect and aberrant lysosomal targeting reduces ARSA activity to ∼8% of normal^39^. Similarly, the ARSA missense variant c.511 G > A (p.Asp171Asn; rs74315466) found at high frequency in our study, which is reported to lie within a second N-glycosylation site^19^, may also compromise lysosomal targeting to affect ARSA activity and the ARSA-PD phenotype. However, since it also occurs in *cis* with the other PD variants, we cannot draw conclusions about its possible direct contribution to phenotype in this study.

Arylsulfatase A catalyses the degradation of sulfatides, a subgroup of glycosphingolipids highly expressed in neural tissue but also found in pancreatic islets of Langerhans where they preserve insulin crystals and monomerize insulin for secretion from beta cells^40^. An explanation for their role in diabetes^22^ and the association between ARSA-PD variants and T2D observed here could be this direct effect on insulin secretion. Indeed, treatment with sulfatides in animal models of T2D enhances glucose-stimulated insulin secretion and improves first-phase insulin response^41^. However, sulfatides are found in many tissues, including kidney^42^, and serum sulfatides are a marker of kidney function^43^ and a biomarker for CVD particularly in ESRD^24^. Sulfatides accumulate in high concentrations in distal convoluted tubules and collecting ducts of the renal medulla in *Arsa* knockout mice^44^, where they play a role in urinary acidification and acid-base homeostasis^42^. It was of interest, therefore, that ARSA-PD variants were more strongly associated with QT for CVD/HTN and CRD/ESRD in non-diabetic individuals in our study, suggesting direct effects on renal disease and associated cardiovascular dysfunction.

Overall our results highlight associations between ARSA-PD variants and traits associated with CVD, CRD/ESRD and T2D in Aboriginal Australians. While this likely relates to multiple pleiotropic effects of sulfatides on metabolic functions, this work highlights sulfatides as a possible avenue for therapeutic intervention in CVD, CRD/ESRD and T2D. More broadly, the use of WES to identify functional ClinVar variants provides important baseline information for diagnosis of rare diseases in Aboriginal Australians^15^, while also demonstrating high frequencies for specific functional variants associated with more complex metabolic disease pathways.

## Methods

### Study Population

As reported^8^, family-based recruitment was from an Australian Aboriginal community of Martu ancestry^45^ from Western Australia. A memorandum of understanding with the community included permission for access to clinical records (e.g. QT and clinical phenotype data, as below) held in a Communicare database at the local Aboriginal Health Service. Ethical approval was obtained from, and all protocols approved by, the Western Australian Aboriginal Health Ethics Committee (WAAHEC; Reference 227 12/12). The study was carried out in accordance with the Declaration of Helsinki Principles, and each participant, or the parent/guardian of individuals <18 years old, signed informed consent forms to participate in the study and provide a DNA sample. DNA was prepared from saliva samples collected into Oragene tubes (DNA Genotek, Ontario, Canada) from 405 consenting family members. All procedures were also carried out in accordance with established institutional standard operating procedures for working with human samples. Following feedback to the community, permission to publish was provided by the Board of the local Aboriginal Health Service, which comprised elders representing the extended families residing in the area.

### Genotype data and WES

Post-QC genotype data were available^8^ for 402 individuals typed on the Illumina 2.5 M Duo Beadchip. These individuals belonged to inter-related extended pedigrees, as depicted in the radial plot which shows hierarchical clustering of estimated pairwise identity-by-descent allele-sharing (Supplementary Fig. S3). Principal component (PC) analysis^8^ demonstrated a degree of introgression of predominantly Caucasian origin, with a tight cluster of 195 individuals of pure Martu ancestry across all age groups. Phased haplotypes for arylsulphatase A (*ARSA*) single nucleotide polymorphisms (SNPs) were generated using PLINK^46^. Association analyses were performed in PLINK^46^ on single SNP or phased haplotype data for 89 cases and 109 controls ≥ 20 years of age using logistic regression under an additive model with 10 PCs as covariates. Single marker association analyses in PLINK aligned closely with results of linear mixed models used in the original genome-wide association analyses^8^ to take account of both family relationships and genetic substructure. WES data were available for 72 individuals (35 pure Martu), for which details of sequence and variant analysis are reported elsewhere^16^. Supplementary Table S5 provides basic demographic data (age, sex; T2D status) for the 391 post-QC individuals used in the Illumina 2.5 M Duo Beadchip analyses^8^, and for the 72 individuals (48 adults aged 52.7 ± 16.3 representing extremes of renal disease, as defined by estimated glomerular filtration rate (eGFR), with/without T2D; 24 minors 12.6 ± 7.7 years included for rare variant discovery) with WES^16^.

### Quantitative and phenotypic traits

Renal disease is monitored in the community by regular measurements of albumin:creatinine ratios (ACR; albumin concentration in milligrams/creatinine concentration in grams), estimated glomerular filtration rates (eGFR) based on serum creatinine measurements, and HTN based on blood pressure measurements. Data for diastolic (DBP) and systolic (SBP) blood pressure (mm Hg), ACR, and calculated eGFR data were extracted from the Communicare database. Clinical cut-offs for HTN were SBP ≥ 140 mm Hg and DBP ≥ 90 mm Hg; for renal function normal (eGFR > 60), moderate (eGFR 30–59), severe (eGFR 15–28), and kidney failure (eGFR < 15); and for ACR as normal (ACR < 30), moderately increased (30–300), severely increased (ACR > 300). Diabetes/pre-diabetes was monitored using HbA1c, with normal (≤6%, equivalent to 42.1 mmol/mol or 7.0 mmol/L); pre-diabetic 6–6.4%; diabetic (≥6.5%). BMI was measured as weight (kg)/height (m^2^).

### Defining pathogenic variants

We used two approaches to identify putative pathogenic variants in WES data. Firstly, all called variants were compared with functional disease-associated variants in ClinVar^14^. ClinVar uses the five clinical significance categories recommended by the American College of Medical Genetics and Genomics^47^ (benign, likely benign, uncertain significance, likely pathogenic and pathogenic). We restricted our comparison to the “pathogenic” or “likely pathogenic” (>90% likelihood of being pathogenic) categories, criteria for which are outlined elsewhere^47^. Frequencies for the disease-causing alleles (as defined in ClinVar) of these variants in our study were compared with the maximum frequency (designated Max-all) reported in either the National Heart, Lung, and Blood Institute (NHLBI) Exome Sequencing Project (ESP)^48^, the 1000 Genomes Project^49^, the Exome Aggregation Consortium (ExAC)^50^ or the Genome Aggregation Database (gnomAD)^50^ databases using GEMINI^51^. In reporting ClinVar variants we have included all variants (Table S1) designated as pathogenic, likely pathogenic, or a risk factor in at least one study. These variants ranged from deleterious variants of high burden that have previously been recorded as pathogenic for rare genetic disorders, to those contributing to quantitative and/or more complex phenotypes such as the cardiovascular and renal disease phenotypes of specific interest to this study. Secondly, we assigned raw and scaled Combined Annotation Dependent Depletion (CADD)^17^ values to all variants called in the WES data and filtered for genes carrying potential deleterious variants. CADD uses 63 different annotations for its combined score^17^. To provide a “normalized” and externally comparable unit, the raw CADD scores for all ~8.6 billion single nucleotide variants (SNVs) of the GRCh37/hg19 reference have been ranked and “PHRED-scaled” such that reference genome SNVs at the 10th-% of CADD scores are assigned to CADD-10, top 1% to CADD-20, top 0.1% to CADD-30, etc^17^. Although CADD scores correlate with annotations of functionality and pathogenicity^17^, there is no hard cut-off for deleteriousness to identify potentially pathogenic variants. The authors of CADD suggest a cut-off between 10 and 20 for scaled CADD scores, possibly at 15 which is the median value for all possible canonical splice site changes and non-synonymous variants^17^. We ranked scaled CADD values internal to our study to determine cut-offs that defined the top 1%, 5% and 10% of deleteriousness, and again compared study-specific putative deleterious allele frequencies with Max-all.

### Gene-set enrichment analysis

In addition to manual inspection of genes carrying putative pathogenic or deleterious alleles, we used the gene-set enrichment tool Enrichr^18^ to analyse gene lists for evidence of enrichment of pathogenic/deleterious variants in multiple genes in pathways influencing the phenotypes of interest. Canonical pathways and gene network analyses based on gene lists were also analysed using QIAGEN’s Ingenuity Pathway Analysis (IPA; QIAGEN Inc., https://www.qiagenbioinformatics.com/products/ingenuity-pathway-analysis)^52^. IPA utilises the Ingenuity Knowledge Base, an extensive database comprising biological pathways and functional annotations derived from the interactions between genes, proteins, complexes, drugs, tissues and disease. Benjamini-Hochberg (BH) correction was applied and −log P_BH-adjusted_ = 1.3 (*P*_BH-adjusted_ = 0.05) taken as the threshold to report results of pathway analyses. Networks were constructed in IPA using the “Connect” option under the “Build” functionality.

### Statistical analysis of QT by genotype data

Differences in QT responses between ARSA rs6151429 genotypes were evaluated using parametric unpaired t-tests with Welch’s correction where between group variances were not different, and non-parametric Mann-Whitney tests where variances were unequal (ACR response data only). For comparison across 4 groups, ordinary one-way analysis of variance (ANOVA) with multiple comparisons and correction for multiple testing were employed, or Kruskal-Wallis tests with multiple comparisons (ACR response data only). Analyses were performed in GraphPad Prism 7.00.

### Availability of Data

Chip-based genotype^8^ and WES^16^ data are available through the European Genome-Phenome Archive (https://www.ebi.ac.uk/ega/dataproviders/EGAO00000000341) by application to study-specific data access committee.

### Ethical approval and informed consent

Ethical approval was obtained from, and all protocols approved by, the Western Australian Aboriginal Health Ethics Committee (WAAHEC; Reference 227 12/12). The study was carried out in accordance with the Declaration of Helsinki Principles, and each participant, or the parent/guardian of individuals <18 years old, signed informed consent forms to participate in the study and provide a DNA sample.

## Electronic supplementary material

Supplementary Information
