# Supplementary material for: Arylsulphatase A Pseudodeficiency (ARSA-PD), hypertension and chronic renal disease in Aboriginal Australians
Source: Sci Rep. 2018 Jul 19;8:10912. doi: 10.1038/s41598-018-29279-9 (PMC6053446; doi:10.1038/s41598-018-29279-9)
Supplement: Supplementary file 1 — Supplementary Information [file 41598_2018_29279_MOESM1_ESM.pdf]

Arylsulphatase A Pseudodeficiency (ARSA-PD), hypertension and chronic renal disease in Aboriginal Australians – SUPPLEMENTARY DATA

SREP-17-47025-T

Dave Tang[1], Michaela Fakiola[2], Genevieve Syn[1], Denise Anderson[1], Heather J. Cordell[3], Elizabeth S. H. Scaman[1], Elizabeth Davis[1,4], Simon J. Miles[5], Toby McLeay[5], Sarra E. Jamieson[1], Timo Lassmann[1] and Jenefer M. Blackwell[1]

[1]Telethon Kids Institute, The University of Western Australia, Subiaco, Western Australia 6008, Australia;

[2] National Institute of Molecular Genetics, Milan, Italy;

[3] Institute of Genetic Medicine, Newcastle University, Newcastle upon Tyne, NE1 3BZ, United Kingdom;

[4] Department of Endocrinology and Diabetes, Princess Margaret Hospital for Children, Subiaco, Western Australia 6008, Australia;

[5] Ngangganawili Aboriginal Health Service, Wiluna, Western Australia 6646, Australia.

Correspondence should be addressed to J.M.B. (jenefer.blackwell@telethonkids.org.au)

List of Supplementary Information

Table S1. Summary of 125 ClinVar functional variants identified in 111 genes from 72 Aboriginal Australian exomes.

Table S2. Summary of 352 CADD-scaled variants identified in 301 genes from 72 Aboriginal Australian exomes.

Table S3. Results of gene set enrichment analysis in Enrichr* using 111 genes with 125 variants classified as pathogenic in ClinVar.

Table S4. Haplotype analysis for association between T2D and sliding windows of 2, 3, 4, 5 and 6 SNP haplotypes across the ARSA gene.

Table S5. Characteristics of subjects for WES and GWAS analyses.

Figure S1. Relationship between ARSA rs6151429 genotypes and HbA1c in the study population.

Figure S2. Relationship between ARSA rs6151429 genotypes and BMI in the study population.

Figure S3. Radial plot showing hierarchical clustering of estimated pairwise identity-by□ descent allele□sharing for the 402 genotyped individuals used in the original GWAS. 5,

Table S1. Summary of 125 ClinVar functional variants identified in 111 genes from 72 Aboriginal Australian exomes. Variants are included if they were recorded as pathogenic, likely pathogenic, or a risk factor for a ClinVar disease or phenotype. Since conflicting interpretations of pathogenicity are recorded in ClinVar, only the disease/phenotype for which pathogenecity/likely pathogenicity/risk factor were recorded are provided in the table. Chrom = chromosome; Start = bp position start of variant; End = bp position end of variant; Ref = reference allele; Alt = alternative allele; Clinvar causal allele = disease associated variant; Gene = gene in which variant is found; SNP ID - rs ID as in DbSNP; Impact = effect of variant; cDNA = change caused by variant at cDNA level; Protein = change caused by variant at protein level (note: there may also be effects in alternatively spliced transcripts not shown here); AC = allele count for the variant allele; Vaf = variant allele frequeny in exomes (descending order of frequency); Max var all = maximum frequency of variant allele in public domain databases (see methods); CADD scaled = scaled CADD score; Clinvar assignment = assignment for pathogenicity of variant; MoI = mode of inheritance (RF = risk factor; AR = autosomal recessive; AD = autosomal dominant; XLR = X-linked recessive; XLD = X-linked dominant; QTL = quantitative trait locus); ClinVar disease name = disease for which ClinVar records the pathogenicity assignment indicated. Bold indicates genes that are present in the top canonical pathways (see Figure 1, main text), and the ARSA variants of specific interest in this study.

Chrom	Start	End	Ref	Alt	Clinvar causal allele	Gene	SNP ID	Impact	cDNA	Protein	AC	Genotype Frequencies			Vaf	Max vaf all	CADD scaled	Clinvar assignment	MoI	Clinvar disease name/Phenotype
												Hom Ref	Het	Hom Var						
chr7	150696110	150696111	T	G	G	NOS3	rs1799983	missense	c.894T>G	p.Asp298Glu	124	1	18	53	0.861	0.930	0.2	risk factor	RF	Coronary artery spasm 1; Alzheimer disease, late-onset; Hypertension, pregnancy-induced; Hypertension resistant to conventional therapy; Ischemic heart disease; Ischemic stroke.
chr16	27356202	27356203	A	G	G	IL4R	rs1805010	missense	c.223A>G	p.Ile75Val	122	2	18	52	0.847	0.516	0.02	protective, pathogenic	RF	Atopy, resistance to; Acquired immunodeficiency syndrome
chr19	13010519	13010520	A	G	G	GCDH	rs8012	missense	c.1250A>G	p.Gln417Arg	96	9	30	33	0.667	0.856	0.53	likely-pathogenic	AR	Glutaric aciduria, type_1
chr1	171076965	171076966	G	A	A	FMO3	rs2266782	splice variant	c.472G>A	p.Glu158Lys	93	14	23	35	0.646	0.496	10.12	pathogenic	AR	Trimethylaminuria
chr19	13010642	13010643	G	T	T	GCDH	rs9384	3' UTR	c.*288G>T	NA	88	14	28	30	0.611	0.442	1.17	likely-pathogenic	AR	Glutaric aciduria, type_1
chrX	105278360	105278361	C	A	A	SERPINA7	rs1804495**	missense	c.909G>T	p.Leu303Phe	50	5	26	12	0.581	0.299	14.9	pathogenic	QTL XLR	Thyroxine-binding globulin, variant P
chr9	136301981	136301982	C	G	G	ADAMTS13	rs2301612	missense	c.1342C>G	p.Gln448Glu	75	16	37	19	0.521	0.511	0.69	pathogenic	AR	Upshaw-Schulman syndrome
**chr22**	**51063476**	**51063477**	**A**	**G**	**G**	**ARSA**	**rs6151429**	**3' UTR**	**c.*96A>G**	**NA**	**68**	**18**	**40**	**14**	**0.472**	**0.120**	**11.25**	**pathogenic**		**Arylsulfatase A pseudodeficiency**
chrX	105279367	105279368	G	A	A	SERPINA7	rs2234036**	missense	c.631G>A	p.Ala211Thr	41	9	27	7	**0.477**	0.034	12.14	pathogenic	QTL XLR	Thyroxine-binding globulin, variant A
**chr22**	**51065434**	**51065435**	**G**	**A**	**A**	**ARSA**	**rs74315466**	**missense**	**c.511G>A**	**p.Asp171Asn**	**64**	**21**	**38**	**13**	**0.444**	**0.003**	**6.16**	**pathogenic**	**QTL**	**Arylsulfatase A pseudodeficiency**
chr14	21790039	21790040	G	T	T	RPGRIP1	rs10151259	missense	c.1639G>T	p.Ala547Ser	64	24	32	16	0.444	0.245	13.8	pathogenic	RF	Cone-rod dystrophy 13
chr12	121176082	121176083	G	A	A	ACADS	rs1799958	missense	c.625G>A	p.Gly209Ser	64	21	38	13	0.444	0.379	25.1	pathogenic	AR	Deficiency of butyryl-CoA dehydrogenase
chr5	35861067	35861068	T	C	C	IL7R	rs1494558	missense	c.197T>C	p.Ile66Thr	63	23	35	14	0.438	0.763	0.02	pathogenic	AR	Severe combined immunodeficiency, autosomal recessive, T cell-negative, B_cell-positive, NK_cell-positive
chr19	49469086	49469087	T	C	C	FTL	rs2230267	synonymous	c.163T>C	p.Leu55=	62	21	40	11	0.431	0.543	11.91	likely-pathogenic	RF	sporadic abdominal aortic aneurysm
chr10	70645375	70645376	A	C	C	STOX1	rs10509305	missense	c.1824A>C	p.Glu608Asp	57	28	31	13	0.396	0.316	0	pathogenic	RF	Preeclampsia/eclampsia 4
chr4	187158033	187158034	G	A	A	KLKB1	rs3733402	missense	c.428G>A	p.Ser143Asn	51	32	29	11	0.354	0.775	0.02	pathogenic	AR	Prekallikrein deficiency
chr6	151936676	151936677	G	A	A	CCDC170/ES	rs6929137	missense	c.1810G>A	p.Val604Ile	48	33	30	9	0.333	0.503	10.5	likely-pathogenic	AR	Estrogen resistance
**chr19**	**45411940**	**45411941**	**T**	**C**	**C**	**APOE**	**rs429358**	**missense**	**c.388T>C**	**p.Cys130Arg**	**47**	**33**	**31**	**8**	**0.326**	**0.273**	**0.01**	**pathogenic**	**RF**	**Familial type 3 hyperlipoproteinemia; APOE4(-)-Freiburg**
**chr5**	**176520242**	**176520243**	**G**	**A**	**A**	**FGFR4**	**rs351855**	**missense**	**c.1162G>A**	**p.Gly388Arg**	**46**	**29**	**40**	**3**	**0.319**	**0.463**	**19.01**	**pathogenic**	**RF**	**Cancer progression and tumor cell motility**
chr7	117188681	117188684	GTT	G	G	CFTR	rs727504486	intronic	c.1210-12_1210-6T[5]	NA	36*	26	29	8	0.290	0.066	None	pathogenic, risk factor	AR, AD	Congenital bilateral absence of the vas deferens; Cystic_fibrosis; Bronchiectasis with or without elevated sweat chloride 1
chr20	4680250	4680251	A	G	G	PRNP	rs1799990	missense	c.385A>G	p.Met129Val	41	38	27	7	0.285	0.415	11.91	risk factor	RF	Prion disease; Alzheimer disease, early-onset; Aphasia, primary progressive
chr1	231408090	231408091	A	G	G	GNPAT	rs11558492	missense	c.1556A>G	p.Asp519Gly	39	39	27	6	0.271	0.213	11.88	pathogenic (also reported likely benign)	AR	Rhizomelic chondrodysplasia punctata type 2
**chr7**	**138417790**	**138417791**	**A**	**G**	**G**	**ATP6V0A4**	**rs3807153**	**missense**	**c.1739T>C**	**p.Met580Thr**	**38**	**40**	**26**	**6**	**0.264**	**0.213**	**17.15**	**pathogenic**	**AR**	**Renal tubular acidosis, distal, autosomal recessive**
chr11	66328094	66328095	T	C	T	ACTN3	rs1815739	missense	c.1729C>T	p.Arg577Ter	36	39	30	3	0.250	0.115	12.2	pathogenic	QTL, RF	ACTN3 deficiency

chr3	39307161	39307162	G	A	A	CX3CR1	rs3732378	missense	c.841G>A	p.Val281Ile	35	41	27	4	0.243	0.172	11.03	pathogenic, risk factor	RF	Human immunodeficiency virus type 1, rapid progression to AIDS; Coronary artery disease, resistance to; Macular Degeneration, Age-related, 12
chr3	39307255	39307256	C	T	T	CX3CR1	rs3732378	missense	c.841G>A	p.Val281Ile	34	41	28	3	0.236	0.285	0.01	pathogenic, risk factor	RF	Human immunodeficiency virus type 1, rapid progression to AIDS; Coronary artery disease, resistance to; Macular Degeneration, Age-related, 12
chr18	55238819	55238820	A	G	G	FECH	rs2272783	intronic	c.315-48T>C	NA	34	42	26	4	0.236	0.376	1.97	pathogenic	AR	Erythropoietic protoporphyria; Erythema
chr5	150227997	150227998	C	T	T	IRGM	rs10065172	synonymous	c.313C>T	p.Leu105=	34	41	28	3	0.236	0.502	2.48	pathogenic	RF	Inflammatory bowel disease 19
chr17	78087040	78087041	G	A	A	GAA	rs1800309	missense	c.2065G>A	p.Glu689Lys	31	46	21	5	0.215	0.310	9.1	pathogenic	AR	Acid alpha-glucosidase, allele 4
chr16	69745144	69745145	G	A	A	NQO1	rs1800566	missense	c.559C>T	p.Pro187Ser	26*	45	14	6	0.200	0.457	23.7	risk factor, pathogenic	RF	Benzene toxicity; Leukemia, post-chemotherapy; Breast cancer, post-chemotherapy poor survival
chr6	49580246	49580247	C	T	T	RHAG	rs16879498	missense	c.808G>A	p.Val270Ile	28	45	26	1	0.194	0.135	28.2	pathogenic	AD	Rh-null hemolytic anemia, regulator type
chr9	116153890	116153891	C	G	G	ALAD	rs1800435	missense	c.177G>C	p.Lys59Asn	27	48	21	3	0.188	0.159	2.24	pathogenic	AR	Aminolevulinate_dehydratase, ALAD*1/ALAD*2 polymorphism
chr22	18901003	18901004	G	A	G	PRODH	rs450046	missense	c.1562A>G	p.Gln521Arg	25	49	21	2	0.174	0.035	11.32	pathogenic, risk factor	AR, AD	Proline dehydrogenase deficiency; Schizophrenia 4
chr19	35786867	35786868	C	T	G	MAG	rs2301600	missense	c.399C>G	p.Ser133Arg	24	49	22	1	0.167	0.423	8.6	pathogenic	AR	Spastic paraplegia 75, autosomal recessive
chr4	24801833	24801834	C	G	G	SOD3	rs1799895	missense	c.691C>G	p.Arg231Gly	21*	53	13	4	0.150	0.141	15.48	pathogenic	QTL, RF	Superoxide dismutase, elevated extracellular
chr3	38645419	38645420	T	C	C	SCN5A	rs1805124	missense	c.1535C>T<br>c.1673A>G	p.Thr512Ile<br>p.His558Arg	21	54	15	3	0.146	0.309	0.35	pathogenic haplotype	AD	Progressive familial heart block type 1A
chr11	113270827	113270828	G	A	A	ANKK1	rs1800497	missense	c.2137G>A	p.Glu713Lys	21	54	16	2	0.146	0.489	0.01	pathogenic, drug-response	RF	Dopamine receptor d2, reduced brain density; bupropion response-Efficacy; ethanol-, clozapine-, olanzapine-, antipsychotics-, risperidone-responses Toxicity/ADR
chr11	48145374	48145375	A	C	C	PTPRJ	rs1566734	missense	c.827A>C	p.Gln276Pro	20	55	14	3	0.139	0.319	10.21	pathogenic	RF	Carcinoma of colon
chr17	42338944	42338945	T	C	C	SLC4A1	rs5036	missense	c.166A>G	p.Lys56Glu	17	56	15	1	0.118	0.133	0.02	pathogenic	polymorphism	Band 3 memphis
chr10	99509250	99509251	G	T	T	ZFYVE27	rs35077384	missense	c.572G>T	p.Gly191Val	13	59	13	0	0.090	0.086	14.71	pathogenic	AD	Spastic paraplegia 33, autosomal dominant
chr16	16251598	16251599	C	T	T	ABCC6	rs2238472	missense	c.3803G>A	p.Arg1268Gln	12	60	12	0	0.083	0.313	11.76	pathogenic	AR	Pseudoxanthoma elasticum
chr5	167937605	167937606	C	T	T	RARS	rs139644798	missense	c.1367C>T	p.Ser456Leu	11	62	9	1	0.076	0.002	17.85	pathogenic	AR	Leukodystrophy, hypomyelinating, 9
chr11	36615074	36615075	G	A	A	RAG2	rs35691292	missense	c.644C>T	p.Thr215Ile	11	61	11	0	0.076	0.033	12.48	pathogenic	AR	Severe combined immunodeficiency, B cell-negative
chr11	62381082	62381083	T	TG	TG	ROM1	rs527236104	frameshift	c.331dupG	p.Leu114Alafs	10	63	8	1	0.069	0.037	None	pathogenic	AD,AR	Retinitis pigmentosa 7, digenic
chr3	10331456	10331457	G	T	T	GHRL	rs696217	missense	c.178C>A	p.Leu60Met	9	63	9	0	0.063	0.193	13.66	pathogenic, risk factor	AD, AR	Obesity, age at onset; Metabolic syndrome
chr11	89017960	89017961	G	A	A	TYR	rs1126809	missense	c.1205G>A	p.Arg402Gln	9	63	9	0	0.063	0.281	29.5	pathogenic, risk factor	AR, AD, RF	Oculocutaneous albinism types 1 and 1B; Waardenburg syndrome 2 and ocular albinism, digenic; Cutaneous malignant melanoma 8
chr20	23618426	23618427	C	T	T	CST3	rs1064039	missense	c.73G>A	p.Ala25Thr	9	63	9	0	0.063	0.364	10.97	pathogenic	RF	Age-related macular degeneration 11
chr2	234669143	234669144	G	A	A	UGT1A8	rs4148323	missense	c.211G>A	p.Gly71Arg	8	64	8	0	0.056	0.154	12.79	pathogenic, likely-pathogenic	QTL, RF	Hyperbilirubinemia transient familial neonatal; Bilirubin, serum level, quantitative trait locus 1; Hyperbilirubinemia
chr10	69966659	69966660	G	A	A	MYPN	rs199476416	missense	c.3793G>C	p.Ala1265Pro	7	65	7	0	0.049	0.000	19.95	likely-pathogenic	RF	Dilated cardiomyopathy
chr10	88477866	88477867	C	T	T	LDB3	rs145983824	missense	c.1823C>T	p.Pro608Leu	7	65	7	0	0.049	0.000	26.7	pathogenic	AD	Familial hypertrophic cardiomyopathy 24

chr9	137642653	137642654	G	A	A	COL5A1	rs61735045	missense	c.1588G>A	p.Gly530Ser	7	65	7	0	0.049	0.047	14.49	pathogenic	AD	Ehlers-Danlos syndrome, classic_type
chr14	75514137	75514138	C	A	A	MLH3	rs28756990	missense	c.2221G>T	p.Val741Phe	7	65	7	0	0.049	0.052	6.4	pathogenic	RF	Endometrial carcinoma; Hereditary nonpolyposis colorectal cancer type 7
chr6	26093140	26093141	G	A	A	HFE	rs1800562	missense	c.845G>A	p.Cys282Tyr	6	66	6	0	0.042	0.064	17.19	pathogenic, risk factor	AR, AD	Hemochromatosis type 1; Porphyria cutanea tarda; Porphyria variegata,; Hemochromatosis, juvenile, digenic; Alzheimer disease; Microvascular complications diabetes 7; Hereditary cancer-predisposing syndrome; Hereditary hemochromatosis
chr17	12915008	12915009	G	A	A	ELAC2	p.Ser217Leu	missense	c.650C>T	p.Ser217Leu	6	66	6	0	0.042	0.315	9.67	pathogenic	RF	Prostate cancer, hereditary, 2
**chr19**	**45412078**	**45412079**	**C**	**T**	**T**	**APOE**	**rs7412**	**missense**	**c.526C>T**	**p.Arg176Cys**	**4***	**53**	**2**	**1**	**0.036**	**0.185**	**15.82**	**pathogenic, drug-response**	**RF**	**APOE2 Isoforms; Familial type 3 hyperlipo-proteinemia; Apolipo-proteinemia E1; atorvastatin response-efficacy**
chr4	88533539	88533540	A	T	T	DSPP	rs36094464	missense	c.202A>T	p.Arg68Trp	5	67	5	0	0.035	0.381	13.57	pathogenic	AD	Dentinogenesis imperfecta-Shield's type II
chrX	13764944	13764946	CA	C	C	OFD1	rs398122866 **	frameshift	c.688_705del18	p.Ile230_Lys235	2	41	2	0	0.023	0.016	None	pathogenic	XLD, XLR	Oral-facial-digital syndrome; Joubert syndrome 10
chr12	10271086	10271087	A	C	C	CLEC7A	rs16910526	stop_gained	c.714T>G	NA	4	68	4	0	0.028	0.096	14.03	pathogenic	AR	Familial chronic mucocutaneous candidiasis
chr1	115236056	115236057	G	A	A	AMPD1	rs17602729	stop_gained	c.133C>T	NA	4	68	4	0	0.028	0.131	29.9	pathogenic	AR	Muscle AMP deaminase deficiency
chr21	43906537	43906538	C	T	T	RSPH1	rs587777060	missense	c.308G>A	p.Gly103Asp	3	69	3	0	0.021	0.000	18	pathogenic	AR	Primary ciliary dyskinesia 24
**chr11**	**116701559**	**116701560**	**G**	**A**	**A**	**APOC3**	**rs147210663**	**missense**	**c.127G>A**	**p.Ala43Thr**	**3**	69	3	0	**0.021**	**0.009**	**21.3**	**protective, pathogenic**	**QTL**	**Coronary heart disease; Hyper-alphalipoproteinemia 2**
chr7	117149146	117149147	G	A	A	CFTR	rs1800076	missense	c.224G>A	p.Arg75Gln	3	69	3	0	0.021	0.031	34	pathogenic	AD	Hereditary pancreatitis
chr1	158624527	158624528	G	T	T	SPTA1	rs35948326	missense	c.2909C>A	p.Ala970Asp	3	69	3	0	0.021	0.050	10.91	pathogenic	AR	Spherocytosis, type 3, autosomal recessive
chr3	121507230	121507231	A	T	T	IQCB1	rs1141528	missense	c.1178T>A	p.Ile393Asn	3	69	3	0	0.021	0.136	18.02	pathogenic	AR	Nephronophthisis, end stage renal disease, Senior-Loken syndrome 5
chr8	11614574	11614575	A	G	G	GATA4	rs3729856	missense	c.1129A>G	p.Ser377Gly	3	69	3	0	0.021	0.140	11.91	likely-pathogenic	not known	not_provided***
**chr17**	**45360729**	**45360730**	**T**	**C**	**C**	**ITGB3**	**rs5918**	**missense**	**c.176T>C**	**p.Leu59Pro**	**3**	69	3	0	**0.021**	**0.155**	**6.67**	**pathogenic, risk factor**	**AR**	**Thrombocytopenia, neonatal alloimmune; Posttransfusion purpura; Myocardial infarction; Fracture, hip**
chr12	122295334	122295335	A	G	A	HPD	rs1154510	missense	c.97G>A	p.Ala33Thr	2	70	2	0	0.014	0.035	8.78	pathogenic	AD	4-Alpha-hydroxyphenylpyruvate hydroxylase deficiency
chr21	35821706	35821707	C	T	T	KCNE1	rs74315445	missense	c.226G>A	p.Asp76Asn	2	70	2	0	0.014	0.000	15.56	pathogenic, likely-pathogenic	AR	Jervell and Lange-Nielsen syndromes 1 and 2; Long QT syndrome 5; Cardiovascular phenotype
chr10	99361675	99361676	C	T	T	HOGA1	rs796052086	stop_gained	c.763C>T	p.Arg255Ter	2	70	2	0	0.014	0.000	38	pathogenic	not known	Primary hyperoxaluria, type III
chr3	15685832	15685833	G	A	A	BTD	rs104893687	missense	c.235C>T	p.Arg79Cys	2	70	2	0	0.014	0.000	14.17	pathogenic	AR	Biotinidase deficiency
chr2	220075520	220075521	C	T	T	ABCB6	rs148211042	missense	c.2168G>A	p.Arg723Gln	2	70	2	0	0.014	0.001	37	pathogenic	AD	Pseudohyperkalemia, familial, 2, due to red cell leak
chr14	88411974	88411975	C	T	T	GALC	rs200378205	missense	c.1592G>A	p.Arg531His	2	70	2	0	0.014	0.002	17.14	pathogenic	AR	Galactosylceramide beta-galactosidase deficiency
chr13	52520507	52520508	G	A	A	ATP7B	rs41292782	missense	c.2972C>T	p.Thr991Met	2	70	2	0	0.014	0.002	20.8	likely-pathogenic	AR	Wilson disease
chr17	16219999	16220000	T	C	C	PIGL	rs145303331	missense	c.500T>C	p.Leu167Pro	2	70	2	0	0.014	0.003	15.3	pathogenic	AR	Zunich neuroectodermal syndrome; Coloboma, Congenital Heart Disease; Ichthyosiform Dermatosis, Intellectual Disability, and Ear Anomalies (CHIME) Syndrome

chr1	94508968	94508969	G	A	A	ABCA4	rs61751374	missense	c.3113C>T	p.Ala1038Val	2	70	2	0	0.014	0.004	11.9	pathogenic	AR	Stargardt disease 1; Cone-rod dystrophy 3
chr7	40498795	40498796	C	T	T	SUGCT	rs137852860	missense	c.1006C>T	p.Arg336Trp	2	70	2	0	0.014	0.009	13.85	pathogenic	AR	Glutaryl-CoA oxidase deficiency
chr11	128709125	128709126	A	G	G	KCNJ1	rs59172778	missense	c.1013T>C	p.Met338Thr	2	70	2	0	0.014	0.012	8.14	pathogenic	AR	Bartter syndrome, type_2, antenatal
chr12	6458349	6458350	A	G	G	SCNN1A	rs5742912	missense	c.1477T>C	Trp493Arg	2	70	2	0	0.014	0.026	20.1	,pathogenic	AD	Bronchiectasis with or without elevated sweat chloride 2
chr20	18505199	18505200	G	T	T	SEC23B	rs36023150	missense	c.490G>T	p.Val164Leu	2	70	2	0	0.014	0.034	33	pathogenic	AD	Cowden syndrome 7
chr2	167141108	167141109	G	T	T	SCN9A	rs41268673	missense	c.1828C>A	p.Pro610Thr	2	70	2	0	0.014	0.035	7.2	pathogenic	AD	Primary erythromelalgia
chr2	215813330	215813331	C	T	T	ABCA12	rs726070	missense	c.6139G>A	p.Asp2047Asn	2	70	2	0	0.014	0.036	12.74	pathogenic	AR	Autosomal recessive congenital ichthyosis 4B
chr17	12899901	12899902	C	T	T	ELAC2	rs5030739	missense	c.1621G>A	p.Ala541Thr	2	70	2	0	0.014	0.050	10.37	pathogenic	not known	Prostate cancer, hereditary, 2
chr1	156146639	156146640	G	A	A	SEMA4A	rs41265017	missense	c.2138G>A	p.Arg713Gln	2	70	2	0	0.014	0.064	9.99	pathogenic	AR,AD	Retinitis pigmentosa 35
chr10	54531241	54531242	G	A	A	MBL2	rs5030737	missense	c.154C>T	p.Arg52Cys	2	70	2	0	0.014	0.076	17.29	pathogenic	AD	Mannose-binding protein deficiency
chr16	89986143	89986144	C	T	T	MC1R	rs1805008	missense	c.478C>T	p.Arg160Trp	2	70	2	0	0.014	0.081	13.77	pathogenic, risk factor	not known	Skin/hair/eye pigmentation 2, red hair/fair skin; Increased analgesia from kappa-opioid receptor agonist; Oculocutaneous Albinism, TYPE II
**chr21**	**46931108**	**46931109**	**G**	**A**	**A**	**COL18A1**	**rs12483377**	**missense**	**c.4309G>A**	**p.Asp1437Asn**	**2**	70	2	0	**0.014**	**0.149**	**21.5**	**pathogenic**	**AR**	**Knobloch syndrome 1**
chr9	34649441	34649442	A	G	G	GALT	rs2070074	missense	c.940A>G	p.Asn314Asp	2	70	2	0	0.014	0.187	5.94	pathogenic	AR	Deficiency of UDPglucose-hexose-1-phosphate uridylyltransferase
chr10	54531234	54531235	C	T	T	MBL2	rs1800450	missense	c.161G>A	p.Gly54Asp	2	70	2	0	0.014	0.219	20.6	pathogenic	AD	Mannose-binding protein deficiency
chr17	72745312	72745313	C	G	G	SLC9A3R1	rs35910969	missense	c.328C>G	p.Leu110Val	1	71	1	0	0.008	0.037	12.92	pathogenic	AD	Nephrolithiasis/osteoporosis, hypophosphatemic, 2
chr11	2930439	2930440	G	A	A	SLC22A18	rs78838117	missense	c.257G>A	p.Arg86His	1	71	1	0	0.007	0.223	5.83	pathogenic	not known	Rhabdomyosarcoma, somatic
chr5	73981269	73981270	T	C	T	HEXB	rs820878	missense	c.185C>T	p.Ser62Leu	1	71	1	0	0.007	0.019	3.14	pathogenic	AR	Sandhoff disease, infantile type
chr4	3494850	3494851	G	GC	GC	DOK7	rs761899995	frameshift	c.*359dupG	p.Ala380Glyfs	1	71	1	0	0.007	0.000	None	pathogenic	AR	Myasthenia, limb-girdle, familial
chr2	128412066	128412067	G	A	A	LIMS2	rs768056213	missense	c.275C>T	p.Pro92Leu	1	71	1	0	0.007	0.000	31	pathogenic	AR	Muscular dystrophy, limb-girdle, type 2W
chr16	30768034	30768035	G	A	A	PHKG2	rs572115942	missense	c.926G>A	p.Arg309Gln	1	71	1	0	0.007	0.000	17.53	pathogenic	AR	Mauriac syndrome
chr16	89813255	89813256	T	C	C	FANCA	rs574034197	missense	c.3391A>G	p.Thr1131Ala	1	71	1	0	0.007	0.000	20.1	pathogenic	AR	Fanconi anemia
chr16	84050849	84050850	T	G	G	SLC38A8	rs139373929	missense	c.848A>C	p.Asp283Ala	1	71	1	0	0.007	0.001	20.3	likely-pathogenic	not known	Complies with GeneDX Variant Classification
chr8	100115203	100115204	C	T	T	VPS13B	rs144539572	stop_gained	c.436C>T	p.Arg146Ter	1	71	1	0	0.007	0.001	36	likely-pathogenic	AR	Cohen syndrome
chr1	215853719	215853720	T	C	C	USH2A	rs397517978	splice acceptor	c.12067-2A>G	NA	1	71	1	0	0.007	0.001	16.52	pathogenic	AR	Retinitis pigmentosa 3; Usher syndrome, type 2A
chr1	147231060	147231061	C	A	A	GJA5	rs121434557	missense	c.286G>T	p.Ala96Ser	1	71	1	0	0.007	0.001	27.9	pathogenic	AD	Atrial fibrillation, familial, 11
chr2	179473056	179473057	C	T	T	TTN	rs559590585	missense	c.52553G>A	:c.44849G>A	1	71	1	0	0.007	0.001	14.98	likely-pathogenic	not known	Complies with ACMG Guidelines, 2015
chr16	46723618	46723619	T	C	C	ORC6	rs146795505	start_lost	c.2T>C	p.Met1Thr	1	71	1	0	0.007	0.002	8.98	pathogenic	AR	Meier-Gorlin syndrome 3
chr10	12154928	12154929	G	A	A	DHTKD1	rs117225135	missense	c.2185G>A	p.Gly729Arg	1	71	1	0	0.007	0.003	19.26	pathogenic	AR	2-aminoadipic_2-oxoadipic aciduria
chr3	8787312	8787313	C	G	G	CAV3	rs116840776	missense	c.216C>G	p.Cys72Trp	1	71	1	0	0.007	0.003	14.52	pathogenic	AR,AD	Limb-girdle muscular dystrophy, type 1C
chr1	171076951	171076952	C	T	T	FMO3	rs72549326	missense	c.458C>	p.Pro153Leu	1	71	1	0	0.007	0.003	18.19	pathogenic	AR	Trimethylaminuria
chr1	94473286	94473287	G	A	A	ABCA4	rs28938473	missense	c.5908C>T	p.Leu1970Phe	1	71	1	0	0.007	0.006	26.7	pathogenic, likely-pathogenic	AR	Stargardt disease_1
chr13	37583419	37583420	G	C	C	EXOSC8	rs36027220	missense	c.815G>C	p.Ser272Thr	1	71	1	0	0.007	0.006	12.03	pathogenic	AR	Pontocerebellar hypoplasia, type 1c
chr2	238280503	238280504	C	T	T	COL6A3	rs146092501	missense	c.4156G>A	p.Glu1386Lys	1	71	1	0	0.007	0.008	17.41	likely-pathogenic	AR,AD	Ullrich congenital muscular dystrophy 1
chr1	94517253	94517254	C	G	G	ABCA4	rs76157638	missense	c.2588G>C	p.Gly863Ala	1	71	1	0	0.007	0.008	23.8	pathogenic	AR	Stargardt disease 1
chr5	151202475	151202476	C	T	T	GLRA1	rs116474260	missense	c.1108G>A	p.Gly370Ser	1	71	1	0	0.007	0.016	15.15	pathogenic	AR,AD	Hyperekplexia hereditary
**chr14**	**94844946**	**94844947**	**C**	**T**	**T**	**SERPINA1**	**rs28929474**	**missense**	**c.1096G>A**	**p.Glu366Lys**	**1**	71	1	0	**0.007**	**0.018**	**21.9**	**pathogenic**	**AR**	**Alpha-1-antitrypsin deficiency; FRAXE**
**chr8**	**19813528**	**19813529**	**A**	**G**	**G**	**LPL**	**rs268**	**missense**	**c.953A>G**	**p.Asn318Ser**	**1**	71	1	0	**0.007**	**0.020**	**13.78**	**pathogenic**	**AD**	**Hyperlipidemia, familial combined**
chr1	227170647	227170648	C	T	T	COQ8A	rs41303129	synonymous	c.993C>T	p.Phe331=	1	71	1	0	0.007	0.029	12.62	pathogenic	AR	Coenzyme Q10 deficiency, primary, 4
chr5	35072711	35072712	T	G	G	PRLR	rs72478580	missense	c.508A>C	p.Ile170Leu	1	71	1	0	0.007	0.032	1.86	pathogenic	AD	Multiple_fibroadenomas of the breast
chr1	63872031	63872032	T	C	C	ALG6	rs35383149	missense	c.391T>C	p.Tyr131His	1	71	1	0	0.007	0.041	15.07	pathogenic	AR	Congenital disorder of glycosylation type 1C
chr1	94512564	94512565	C	T	T	ABCA4	rs1801581	missense	c.2828G>A	p.Arg943Gln	1	71	1	0	0.007	0.042	11.01	pathogenic	AR	Stargardt disease 1

chr3	15686692	15686693	G	C	C	BTD	rs13078881	missense	c.1330G>C	p.Asp444His	1	71	1	0	0.007	0.043	16.02	pathogenic	AR	Biotinidase deficiency
chr10	72360386	72360387	G	A	A	PRF1	rs35947132	missense	c.272C>T	p.Ala91Val	1	71	1	0	0.007	0.048	19.66	pathogenic	AR	Hemophagocytic lymphohistiocytosis, familial, 2
**chr11**	**116691633**	**116691634**	**C**	**A**	**A**	**APOA4**	**rs5110**	**missense**	**c.1140G>T**	**p.Gln380His**	**1**	71	1	0	**0.007**	**0.083**	**4.51**	**pathogenic**	**QTL**	**Apoliprotein A-IV polymorphism, APOA4*1/APOA4*2**
chr4	15513003	15513007	GGAA	G	G	CC2D2A	rs386833764	inframe deletion	c.685_687 delGAA	p.Glu229del	1	71	1	0	0.007	0.097	None	likely-pathogenic	AR	Meckel syndrome type 6; Meckel-Gruber syndrome; Familial aplasia of the vermis
**chr2**	**44066246**	**44066247**	**G**	**C**	**C**	**ABCG8**	**rs11887534**	**missense**	**c.55G>C**	**p.Asp19His**	**1**	71	1	0	**0.007**	**0.099**	**14.5**	**pathogenic**	**not known**	**Gallbladder disease 4**
chr3	45814093	45814094	G	A	A	SLC6A20	rs17279437	missense	c.596C>T	p.Thr199Met	1	71	1	0	0.007	0.102	18.49	pathogenic	AD	Hyperglycinuria; Iminoglycinuria, digenic
chr6	26091178	26091179	C	G	G	HFE	rs1799945	missense	c.187C>G	p.His63Asp	1	71	1	0	0.007	0.172	12.44	pathogenic, risk factor	AR	Hemochromatosis type 1; Microvascular complications of diabetes 7
chr2	190925076	190925077	T	C	C	MSTN	rs1805086	missense	c.458A>G	p.Lys153Arg	1	71	1	0	0.007	0.219	8.86	pathogenic	not known	Muscle hypertrophy
chrX	119760628	119760629	A	T	T	C1GALT1C1	rs17261572**	missense	c.393T>A	p.Asp131Glu	1	42	1	0	0.012	0.287	0.41	pathogenic	not known	Polyagglutinable erythrocyte syndrome

* The denominators for these variants were N=126, N=130, N=140, N=112, respectively. For all other variants the denominator was N=144 (i.e. 2N for 72 individuals). **For X-linked genes the genotype frequencies and allele frequencies shown are for females. For males, the numbers of apparent homozygotes for the alternative allele were 13, 12, 0, 0 for rs1804495, rs2234036, rs398122866, and rs17261572, respectively. ***The information provided in ClinVar suggests that this GATA4 variant may not be pathogenic for a rare disease single gene disorder given the high frequency in ExAC. Specifically, ClinVar reports that the "germline p.Ser377Gly in exon 6 of GATA4 is not expected to have clinical significance because it is not located within the splice consensus sequence. It has been identified in 14% (927/6596) of European chromosomes, including 67 homozygotes by the Exome Aggregation Consortium (ExAC, http://exac.broadinstitute.org; dbSNP rs3729856)". However, this does not preclude the possibility that this variant may be a risk factor for cardiovascular phenotypes as reported in ClinVar. We have therefore retained this GATA4 variant in Table S1 and in the gene set enrichment and pathway analyses.

Table S2. Summary of 352 CADD-scaled variants identified in 301 genes from 72 Aboriginal Australian exomes. Variants are included if they were in the top 10% of CADD-scaled scores and were present at variant allele frequency (Vaf) >0.2 in the study population, but at maximum Vaf (Max Vaf all) <0.1 in public domain databases (as described in methods, main text; -1 indicates not present; 0 indicated Vaf<0.001). Grey shading indicates cut-off for top 5% of gene CADD-scaled >15.91. Chrom = chromosome; Start = bp position start of variant; End = bp position end of variant; Ref = reference allele; Alt = alternative allele; Gene = gene in which variant is found; SNP ID - rs ID as in DbSNP (where available); Impact = effect of variant; Exon = exon in which variant is found; Codon change = change caused by variant at g/cDNA level; AA change = change caused by variant at protein level (note: there may also be effects in alternatively spliced transcripts not shown here); AA position = position of AA change over total AA length of protein; AC = allele count (N=144); Vaf = variant allele frequeny in exomes; Max var all = maximum frequency of variant allele in public domain databases (see methods); CADD scaled = scaled CADD score (descending order). Bold indicates genes present in the top GO Biological Process 1917b for negative regulation of adiponectin secretion (Enrichr $P_{nominal} = 5.4x10^{-6}$, $P_{adjusted} = 0.019$) .

Chrom	Start	End	Ref	Alt	Gene	SNP ID	Impact	Exon	Codon change	AA change	AA position	AC	Vaf	Max vaf all	CADD scaled
chr1	36056255	36056256	G	A	TFAP2E	rs114404250	missense	6 of 7	cGa/cAa	R/Q	309/442	51	0.354	0.013	37
chr1	226550828	226550829	T	C	PARP1	rs3219145	missense	21 of 23	aAg/aGg	K/R	940/1014	33	0.229	0.090	35
chr22	30684780	30684781	G	T	RP1-130H16.18	rs189378288	intronic				-/480	110	0.764	0.019	33
chr16	15715635	15715636	C	G	KIAA0430	rs35438466	missense	11 of 25	Gtc/Ctc	V/L	534/1430	50	0.347	0.033	33
chr5	170236615	170236616	C	T	GABRP	rs79997355	missense	10 of 11	Cgc/Tgc	R/C	293/440	80	0.556	0.045	32
chr2	152404217	152404218	T	C	NEB	None	missense	132 of182	gAc/gGc	D/G	6731/8525	1	0.311	-1	30
chr3	196735686	196735687	C	T	MFI2	rs17129219	missense	12 of 16	Gcc/Acc	A/T	559/738	32	0.222	0.053	29.2
chr3	197431999	197432000	C	T	KIAA0226	rs112632845	missense	3 of 20	cGg/cAg	R/Q	89/972	64	0.444	0.052	27.9
chr14	65197809	65197810	G	T	PLEKHG3	rs779117999	missense	5 of 15	Gcc/Tcc	A/S	202/1163	35	0.243	0	27.8
chr3	121151783	121151784	G	A	POLQ	rs2306211	missense	29 of 30	gCa/gTa	A/V	2547/2590	38	0.264	0.076	27.8
chr1	46093868	46093869	T	G	GPBP1L1	rs114265929	3' UTR	13/13			-/474	71	0.493	0.032	27.1
chr4	17819694	17819695	G	A	NCAPG	rs77524902	missense	7 of 21	Gca/Aca	A/T	368/1015	33	0.229	0.066	26.9
chr2	9013423	9013424	C	T	MBOAT2	rs34573615	missense	8 of 13	Gtt/Att	V/I	233/520	42	0.292	0.031	26.8
chr11	105845360	105845361	T	C	GRIA4	rs73552647	intronic				-/884	39	0.283	0.096	26.8
chr20	61467841	61467842	A	G	COL9A3	rs140686800	missense	29 of 32	Agc/Ggc	S/G	521/684	30	0.208	0.017	26.1
chr1	156180721	156180722	G	A	SLC25A44	rs141836547	3' UTR	4 of 4			-/314	31	0.215	0.006	25.7
chr1	225590235	225590236	T	A	LBR	rs75904736	3' UTR	14 of 14			-/615	46	0.319	0.017	25.2
chr17	79660594	79660595	T	A	HGS	rs200174222	missense	9 of 10	cTg/cAg	L/Q	226/263	32	0.222	0.001	25
chr12	53091675	53091676	C	T	KRT77	rs668313	missense	2 of 9	cGa/cAa	R/Q	183/578	52	0.361	0.068	24.4
chr12	100166886	100166887	G	T	ANKS1B	rs374274479	intronic				-/1248	30	0.208	0	24.1
chr12	52711548	52711549	G	T	KRT83	rs2857667	stop gained	4 of 9	tgC/tgA	C/*	222/493	1	0.352	0.009	23.8
chrX	2836183	2836184	C	T	ARSD	rs73632976	missense	5 of 10	gGc/gAc	G/D	175/593	29	0.201	0.084	23.6

chr7	100172558	100172559	G	A	LRCH4	rs74987581	3' UTR	18 of 18			-/683	42	0.292	0.071	23.5
chr3	136059460	136059461	G	A	STAG1	rs3821444	intronic				-/1221	83	0.576	0.052	23.4
chr15	37178740	37178741	A	G	MEIS2	rs12898820	downstream				-/470	40	0.278	0.092	23.2
chr4	31147117	31147118	A	C	PCDH7	rs76036989	3' UTR	3 of 3			-/937	46	0.319	0.083	23.1
chr14	76425344	76425345	G	A	TGFB3	rs3917216	3' UTR	7 of 7			-/412	39	0.271	0.009	22.4
chr12	102141012	102141013	C	T	GNPTAB	rs373314316	missense	21 of 21	Gca/Aca	A/T	1234/1256	58	0.403	0	22.3
chr3	16301842	16301843	T	C	DPH3	rs2292615	3' UTR	2 of 2			-/57	34	0.236	0.030	22.3
chr1	225590674	225590675	A	C	LBR	rs16844841	3' UTR	14 of 14			-/615	49	0.340	0.070	22.3
chr3	196743993	196743994	G	A	MFI2	rs2276790	missense	7 of 16	Cgg/Tgg	R/W	294/738	32	0.222	0.075	22.2
chr12	53088524	53088525	G	A	KRT77	rs585664	missense	5 of 9	tCc/tTc	S/F	322/578	52	0.361	0.068	21.3
chr1	170243123	170243124	A	T	LINC01142	rs41314444	non-coding exon	2 of 3				30	0.208	0.030	21.1
chr9	14842657	14842658	C	G	FREM1	rs41298151	missense	10 of 38	gGg/gCg	G/A	465/2179	61	0.469	0.053	20.9
chr1	183622544	183622545	A	G	RGL1	rs115408195	intronic				-/766	44	0.310	0.013	20.8
chr4	2307224	2307225	G	A	ZFYVE28	rs150392759	missense	8 of 13	aCg/aTg	T/M	211/817	62	0.431	0.036	20.7
chr2	160132088	160132089	T	A	WDSUB1	rs16843852	missense	4 of 7	aAa/aTa	K/I	215/384	13	0.211	0	20.6
chr16	69751952	69751953	A	G	NQO1	None	intronic				-/202	39	0.271	-1	20.4
chr15	56208529	56208530	C	G	NEDD4	rs777667904	missense	1 of 25	aGt/aCt	S/T	167/1303	35	0.243	0	20.4
chr2	173885477	173885478	A	G	RAPGEF4	rs73017586	intronic				-/1011	31	0.215	0.032	20.4
chr3	145788332	145788333	A	C	PLOD2	rs13079521	3' UTR	10 of 10			-/418	43	0.303	0.038	20.1
chr19	51537331	51537332	G	A	KLK12	rs61742847	missense	3 of 5	cCg/cTg	P/L	34/111	36	0.250	0.077	19.99
chr1	889237	889238	G	A	NOC2L	rs3828049	missense	8 of 19	gCg/gTg	A/V	271/749	35	0.243	0.088	19.97
chr2	24439047	24439048	A	G	ITSN2	rs3731625	missense	31 of 39	aTt/aCt	I/T	1260/1670	32	0.222	0.090	19.74
chr5	78301325	78301326	A	G	DMGDH	rs41272264	intronic				-/866	29	0.201	0.096	19.73
chr12	6840166	6840167	C	A	COPS7A	rs774622741	missense	8 of 8	Ctc/Atc	L/I	264/275	31	0.215	0.002	19.72
chr19	12154798	12154799	G	C	ZNF878	rs67102109	missense	4 of 4	Cac/Gac	H/D	473/531	49	0.340	0.095	19.58
chr10	101152774	101152775	A	G	CNNM1	rs75555623	3' UTR	11 of 11			-/951	36	0.250	0.071	19.53
chr6	151336662	151336663	G	A	MTHFD1L	rs200271701	missense	2 of 3	cGc/cAc	R/H	38/96	36	0.250	0.003	19.48
chr1	87602420	87602421	A	C	RP5-1052I5.2	None	intronic				-/303	40	0.278	-1	19.46
chr16	75665390	75665391	C	T	KARS	rs370244075	missense	10 of 15	cGg/cAg	R/Q	420/625	36	0.250	0	19.46
chr11	8126154	8126155	C	T	TUB	rs58706265	3' UTR	13 of 13			-/561	35	0.243	0.060	19.42
chr2	173898718	173898719	G	A	RAPGEF4	rs2290376	intronic				-/1011	53	0.368	0.095	19.41
chr6	157714090	157714091	G	C	TMEM242	None	intronic				-/141	52	0.361	-1	19.31
chrX	52891491	52891492	T	C	XAGE3	rs114794367	downstream				-/111	32	0.242	-1	19.24

chrX	2833637	2833638	C	T	ARSD	rs370769167	missense	6 of 10	gGc/gAc	G/D	320/593	29	0.201	0.001	19.13
chr11	59575155	59575156	G	A	MRPL16	rs78416481	intronic				-/86	63	0.438	0.072	19.13
chr16	69748868	69748869	G	A	NQO1	rs1131341	missense	4 of 6	Cgg/Tgg	R/W	139/274	36	0.250	0.062	19.07
chr22	42390886	42390887	C	G	SEP03.	None	3' UTR	10 of 10			-/350	4	0.500	-1	19
chr1	91979446	91979447	C	A	CDC7	None	intronic				-/546	38	0.264	-1	18.95
chr14	59793582	59793583	G	A	DAAM1	rs373571158	intronic				-/1078	34	0.236	-1	18.94
chr6	167366069	167366070	C	G	RNASET2	rs2273746	intronic				-/253	31	0.215	0.085	18.94
chr2	84897500	84897501	A	G	DNAH6	rs17025409	missense	4 of 11	tAt/tGt	Y/C	140/551	36	0.250	0.063	18.9
chr11	8122942	8122943	C	T	TUB	rs2242504	intronic				-/506	44	0.306	0.068	18.75
chr16	58200615	58200616	C	T	CSNK2A2	rs191939499	intronic				-/152	33	0.229	0.082	18.68
chr2	196600148	196600149	T	G	SLC39A10	rs13391047	3' UTR	11 of 11			-/381	61	0.424	0.022	18.65
chr5	148620347	148620348	C	T	ABLIM3	rs77050679	intronic				-/544	34	0.236	0.070	18.65
chr18	52258357	52258358	T	C	DYNAP	rs368060476	intronic				-/158	40	0.278	0.061	18.45
chr11	113283483	113283484	G	C	DRD2	rs1801028	missense	6 of 7	tCc/tGc	S/C	311/443	42	0.292	0.088	18.42
chr19	51832844	51832845	G	A	IGLON5	rs376750388	3' UTR	8 of 8			-/336	39	0.271	-1	18.4
chr10	89507110	89507111	T	C	PAPSS2	rs117022300	3' UTR	12 of 12			-/614	30	0.208	0.095	18.38
chr19	5914649	5914650	G	A	CAPS	rs199930097	missense	3 of 5	Ggg/Agg	G/R	54/189	59	0.410	0.022	18.37
chr15	76193096	76193097	C	T	UBE2Q2	rs75281825	3' UTR	12 of 12			-/340	33	0.229	0.030	18.25
chr10	90034882	90034883	C	T	RNLS	rs72818071	intronic				-/315	31	0.215	0.094	18.25
chr19	49570931	49570932	G	A	KCNA7	rs45483292	3' UTR	2 of 2			-/456	40	0.286	0.094	18.13
chr3	183683144	183683145	A	G	ABCC5	rs3817403	intronic				-/1394	52	0.361	0.074	18.08
chr12	6948774	6948775	G	A	LEPREL2	rs369526248	3' UTR	16 of 16			-/735	77	0.535	0.002	18.02
chr19	4502200	4502201	T	G	HDGFRP2	None	3' UTR	16 of 16			-/675	6	0.402	0	18.01
chr6	57398411	57398412	A	G	PRIM2	rs6937166	intronic				-/346	32	0.235	-1	17.96
chr8	82665329	82665330	G	T	CHMP4C	rs373262776	missense	2 of 5	agG/agT	R/S	74/233	42	0.292	0	17.9
chr14	23826322	23826323	G	T	EFS	rs2231816	3' UTR	5 of 5			-/468	55	0.382	0.087	17.89
chr2	178576702	178576703	C	T	PDE11A	rs78004582	intronic				-/933	48	0.333	0.042	17.88
chr19	51833380	51833381	A	G	IGLON5	rs3752250	3' UTR	8 of 8			-/336	39	0.271	0.011	17.84
chr20	40713243	40713244	T	C	PTPRT	rs73119850	intronic				-/1441	37	0.257	0.059	17.81
chr13	33016554	33016555	T	C	N4BP2L2	None	missense	7 of 10	Aag/Gag	K/E	692/737	45	0.313	0	17.75
chr6	130461075	130461076	C	T	L3MBTL3	rs17633592	3' UTR	22 of 22			-/755	49	0.340	0.053	17.55
chr12	14959469	14959470	A	G	SMCO3	rs11609202	missense	2 of 2	Tgc/Cgc	C/R	49/225	36	0.250	0.067	17.45
chr11	113146538	113146539	A	C	NCAM1	rs373331866	3' UTR	18 of 18			-/848	31	0.215	-1	17.43

chr22	17600312	17600313	C	T	CECR6	rs149370082	missense	2 of 2	Gtc/Atc	V/I	214/223	50	0.347	0.032	17.37
chr15	56285616	56285617	C	T	NEDD4	rs376007297	intronic				-/900	38	0.264	-1	17.32
chr14	59931696	59931697	G	A	GPR135	rs3742646	missense	1 of 1	gCg/gTg	A/V	83/494	5	0.227	0.095	17.3
chr6	138196956	138196957	A	C	TNFAIP3	rs141807543	missense	4 of 9	Atc/Ctc	I/L	207/790	31	0.215	0.001	17.27
chr1	1361594	1361595	G	A	TMEM88B	rs199887791	missense	1 of 2	Gac/Aac	D/N	30/163	40	0.299	0.014	17.27
chr6	57472298	57472299	A	G	PRIM2	rs9396358	intronic				-/346	72	0.500	-1	17.15
chr1	202732016	202732017	T	C	KDM5B	rs374596350	intronic				-/1275	44	0.306	-1	17.14
chr18	59947737	59947738	C	T	KIAA1468	rs189479346	intronic				-/1216	39	0.271	0.005	17.13
chr3	179118889	179118890	C	G	GNB4	None	3' UTR	5 of 5			-/165	32	0.222	-1	17.12
chr1	65095174	65095175	T	C	CACHD1	rs375501473	intronic				-/1274	51	0.354	0	17.12
chr5	126994314	126994315	A	T	CTXN3	rs116673667	3' UTR	3 of 3			-/81	37	0.257	0.035	17.12
chr16	87436763	87436764	A	G	MAP1LC3B	rs1054528	3' UTR	4 of 4			-/125	142	0.986	-1	17.11
chr10	108333818	108333819	T	A	SORCS1	rs72821131	3' UTR	26 of 26			-/1168	17	0.414	-1	17.06
chr14	96797866	96797867	G	C	ATG2B	rs146022217	missense	11 of 42	Cca/Gca	P/A	526/2078	37	0.257	0.076	17.04
chr4	129867227	129867228	C	T	SCLT1	rs112358448	missense	16 of 21	cGt/cAt	R/H	458/688	30	0.208	0.018	17.01
chr6	116442896	116442897	C	T	AL121963.1	rs142463796	missense	3 of 4	tCt/tTt	S/F	93/146	40	0.278	0.042	16.97
chr2	38829561	38829562	G	A	HNRNPLL	rs142358609	intronic				-/275	44	0.306	0.091	16.96
chr1	154243245	154243246	G	T	UBAP2L	rs7554027	3' UTR	26 of 26			-/1087	40	0.513	-1	16.95
chr16	15737274	15737275	G	A	NDE1	rs34560499	5' UTR	1 of 10			-/346	50	0.347	0.033	16.94
chr12	110368803	110368804	C	A	GIT2	rs77766064	3' UTR	19 of 19			-/709	34	0.236	0.064	16.94
chr11	59368212	59368213	C	T	OSBP	rs80049069	intronic				-/807	73	0.507	0.046	16.89
chr3	122474120	122474121	G	C	HSPBAP1	rs61756481	missense	5 of 8	Ctg/Gtg	L/V	243/488	39	0.271	0.077	16.87
chr17	21311770	21311771	A	T	KCNJ12	rs56195865	intronic				-/433	72	0.500	-1	16.86
chr11	46722017	46722018	G	A	ARHGAP1	rs28372918	intronic				-/439	49	0.340	0.098	16.85
chr1	236379116	236379117	A	G	ERO1LB	rs11554612	3' UTR	16 of 16			-/467	49	0.340	0.083	16.84
chr12	54475692	54475693	G	A	RP11-834C11.7	rs75824950	upstream					72	0.500	-1	16.81
chr14	65210363	65210364	G	T	PLEKHG3	rs377386057	missense	4 of 4	caG/caT	Q/H	734/752	30	0.208	0	16.8
chrX	2833604	2833605	C	T	ARSD	rs111939179	stop gained	6 of 10	tGg/tAg	W/*	331/593	29	0.201	0.001	16.78
chr8	23294660	23294661	C	T	ENTPD4	rs146688903	missense	10 of 13	cGa/cAa	R/Q	387/616	35	0.243	0.002	16.74
chr2	179401739	179401740	C	T	TTN	rs55675869	missense	184 of 191	Gtt/Att	V/I	24301/26926	54	0.375	0.071	16.74
chr20	1289584	1289585	A	G	SNPH	rs13163	3' UTR	7 of 7			-/538	44	0.306	0.071	16.69
chr19	51837447	51837448	G	A	VSIG10L	rs181926303	missense	8 of 10	Cgt/Tgt	R/C	806/867	32	0.222	0	16.67
chr16	69390938	69390939	G	A	TERF2	rs201689490	stop gained	2 of 2	Cga/Tga	R/*	31/49	36	0.250	0	16.66

chr3	133191384	133191385	C	A	BFSP2	rs79087781	missense	6 of 7	gCc/gAc	A/D	407/415	47	0.326	0.065	16.65
chr16	28915855	28915856	C	T	RABEP2	rs371094965	3' UTR	13 of 13			-/569	40	0.278	0.031	16.64
chr17	39619185	39619186	G	C	KRT32	rs2604956	missense	6 of 7	gaC/gaG	D/E	371/448	38	0.264	0.094	16.62
chr5	32088499	32088500	C	T	PDZD2	rs3101873	missense	19 of 24	gCc/gTc	A/V	1649/2839	66	0.458	0.043	16.59
chrX	118605022	118605023	T	G	SLC25A5	rs74343587	3' UTR	4 of 4			-/298	61	0.424	0	16.58
chr22	30642643	30642644	G	A	LIF	rs201805161	intronic				-/88	114	0.792	0.030	16.58
chr12	56718421	56718422	T	C	PAN2	rs11171803	synonymous	11 of 26	gcA/gcG	A	557/1202	29	0.201	0.066	16.54
chr1	40776387	40776388	G	A	COL9A2	rs370064150	missense	13 of 14	Ccg/Tcg	P/S	217/226	33	0.229	0.004	16.44
chr12	53564256	53564257	C	T	CSAD	rs80280748	synonymous	8 of 16	gcG/gcA	A	199/493	47	0.326	0.046	16.44
chr3	62578335	62578336	C	T	CADPS	rs17066673	synonymous	7 of 27	gcG/gcA	A	471/1274	30	0.208	0.076	16.4
chr11	14490951	14490952	T	C	COPB1	rs776554381	missense	15 of 22	aAt/aGt	N/S	632/953	34	0.236	0	16.37
chr3	145917627	145917628	C	A	PLSCR4	rs779281159	missense	6 of 9	tGc/tTc	C/F	199/329	29	0.201	0	16.35
chr5	75906922	75906923	C	A	IQGAP2	rs3822530	missense	2 of 5	cCt/cAt	P/H	32/167	82	0.569	0.024	16.35
chr2	179391074	179391075	C	A	TTN	rs72629796	3' UTR	192 of 192			-/27051	54	0.375	0.063	16.35
chr3	47143086	47143087	A	T	SETD2	None	intronic				-/2564	1	0.315	-1	16.32
chr1	78511959	78511960	C	T	GIPC2	rs17101180	missense	1 of 6	tCc/tTc	S/F	61/315	59	0.410	0.096	16.22
chr19	4216876	4216877	G	T	ANKRD24	rs181048027	missense	18 of 22	Gcc/Tcc	A/S	574/1146	30	0.208	0.002	16.19
chrX	2836180	2836181	A	T	ARSD	rs73632975	missense	5 of 10	aTg/aAg	M/K	176/593	29	0.201	0.082	16.17
chr19	8645826	8645827	T	C	ADAMTS10	rs200639529	missense	26 of 26	Agc/Ggc	S/G	1088/1103	43	0.307	0.002	16.15
chr14	23894290	23894291	C	T	MYH7	rs45571436	intronic				-/1935	86	0.597	0.068	16.14
chr17	39577214	39577215	G	A	KRT37	rs8071814	missense	7 of 7	aCg/aTg	T/M	422/449	34	0.236	0.087	16.13
chr17	21320990	21320991	C	T	KCNJ12	rs77190255	3' UTR	3 of 3			-/433	72	0.500	-1	16.1
chr7	98993866	98993867	G	A	PDAP1	rs73711207	3' UTR	6 of 6			-/181	43	0.299	0.079	16.09
chr21	35467125	35467126	C	A	SLC5A3	rs11088273	intronic				-/718	144	1.000	-1	16.05
chr2	119915801	119915802	G	T	C1QL2	rs760809875	missense	1 of 2	gCg/gAg	A/E	15/287	41	0.285	0.003	16.03
chr3	56649255	56649256	C	T	CCDC66	rs61743979	missense	12 of 18	gCg/gTg	A/V	556/948	37	0.257	0.015	16.02
chr7	98993867	98993868	C	G	PDAP1	rs73711208	3' UTR	6 of 6			-/181	43	0.299	0.079	16
chr22	22661317	22661318	T	C	BMS1P20	rs1044437	intronic					47	0.326	-1	15.99
chr21	42554512	42554513	T	C	BACE2	rs572070128	intronic				-/396	2	0.250	0.017	15.96
chr12	109915928	109915929	C	G	UBE3B	rs117721594	intronic				-/1068	34	0.236	0.065	15.96
chr11	59481118	59481119	G	T	OR10V1	rs74398490	missense	1 of 1	gCa/gAa	A/E	67/309	63	0.438	0.046	15.94
chr8	120850729	120850730	G	T	DSCC1	None	intronic				-/393	37	0.257	-1	15.91
chr1	222895946	222895947	A	G	BROX	rs145242855	intronic				-/379	63	0.463	0.031	15.9

chr16	75646261	75646262	C	T	ADAT1	rs151268456	missense	7 of 11	Gtc/Atc	V/I	308/502	36	0.250	0.004	15.89
chr18	5293361	5293362	T	A	ZBTB14	rs369769249	intronic				-/449	30	0.208	0.034	15.86
chr19	50880052	50880053	T	G	NR1H2	rs76217494	5' UTR	2 of 9			-/363	47	0.326	0.061	15.8
chr6	163736265	163736266	T	C	PACRG	rs56157187	3' UTR	5 of 5			-/257	29	0.201	0.076	15.8
chr17	11642230	11642231	A	G	DNAH9	rs61739481	missense	29 of 68	aAg/aGg	K/R	1950/4410	45	0.313	0.093	15.79
chr17	21311744	21311745	T	G	KCNJ12	rs7503781	intronic				-/433	72	0.500	-1	15.78
chr19	51330376	51330377	G	A	KLK15	rs374766999	missense	3 of 4	Cgc/Tgc	R/C	80/171	36	0.250	0	15.78
chr19	51920433	51920434	G	A	SIGLEC10	rs145769059	missense	2 of 11	gCg/gTg	A/V	108/697	65	0.451	0.015	15.76
chr19	13445406	13445407	T	G	CACNA1A	rs28425191	intronic				-/2506	33	0.600	-1	15.66
chrX	153036970	153036971	C	T	PLXNB3	rs138935872	missense	11 of 21	cCg/cTg	P/L	403/988	117	0.813	0.033	15.66
chr10	47000546	47000547	G	T	GPRIN2	rs3127685	3' UTR	1 of 1			-/458	72	0.500	-1	15.64
chr1	206652403	206652404	G	A	IKBKE	rs17021877	missense	9 of 21	Gcc/Acc	A/T	286/631	50	0.347	0.070	15.64
chr16	69680357	69680358	A	G	NFAT5	rs56381721	intronic				-/1531	33	0.232	0.070	15.63
chr22	39813758	39813759	T	C	TAB1	rs35469986	missense	5 of 11	cTt/cCt	L/P	152/504	44	0.306	0.093	15.63
chr17	21320992	21320993	G	T	KCNJ12	rs72842115	3' UTR	3 of 3			-/433	72	0.500	-1	15.62
chr13	74260084	74260085	A	C	KLF12	rs377038470	downstream				-/402	45	0.321	-1	15.61
chr2	68479738	68479739	A	C	PPP3R1	None	intronic				-/189	14	0.636	-1	15.57
chr1	145116410	145116411	A	C	SEC22B	rs782481158	non-coding exon	5 of 5				35	0.243	-1	15.52
chr4	139087707	139087708	T	C	SLC7A11	rs72946076	3' UTR	12 of 12			-/501	35	0.243	0.049	15.52
chr2	56150992	56150993	G	A	EFEMP1	rs3762514	5' UTR	1 of 11			-/493	40	0.278	0.083	15.52
chr6	155139530	155139531	A	G	SCAF8	rs540094163	intronic				-/1337	58	0.403	0.038	15.49
chr18	74728822	74728823	C	T	MBP	rs61742988	missense	1 of 5	Ggt/Agt	G/S	48/160	91	0.632	0.070	15.47
chr17	21320979	21320980	C	G	KCNJ12	rs73313947	3' UTR	3 of 3			-/433	72	0.500	-1	15.46
chr1	11158104	11158105	C	T	EXOSC10	rs62623443	missense	2 of 24	Gaa/Aaa	E/K	74/860	41	0.285	0.091	15.44
chr9	95077469	95077470	T	A	NOL8	rs61742574	missense	6 of 16	aaA/aaT	K/N	411/1099	34	0.236	0.093	15.43
chr14	88945406	88945407	C	G	PTPN21	rs3825676	missense	13 of 19	Ggg/Cgg	G/R	790/1174	68	0.472	0.056	15.42
chr1	36321147	36321148	C	T	AGO4	rs11803314	3' UTR	18 of 18			-/861	37	0.257	0.020	15.4
chr10	55570299	55570300	A	C	PCDH15	rs76528829	intronic				-/1681	45	0.313	0.061	15.4
chr3	124390721	124390722	G	A	KALRN	rs35653635	missense	47 of 59	Ggg/Agg	G/R	2275/2955	39	0.271	0.092	15.4
chr15	79749260	79749261	A	C	KIAA1024	rs79982509	missense	2 of 4	Aat/Cat	N/H	258/916	29	0.201	0.053	15.38
chr9	100778048	100778049	C	T	ANP32B	rs72755412	3' UTR	7 of 7			-/251	46	0.319	-1	15.36
chr5	94114936	94114937	C	G	MCTP1	rs76995120	intronic				-/692	35	0.243	0.098	15.36
chr12	71002792	71002793	A	G	PTPRB	rs76441945	3' UTR	2 of 2			-/103	34	0.236	0.072	15.35

chr10	112570129	112570130	G	C	RBM20	rs12572941	intronic				-/1227	43	0.299	0.097	15.33
chr21	44323719	44323720	C	T	NDUFV3	rs4148974	stop gained	3 of 4	Cga/Tga	R/*	200/473	34	0.236	0.096	15.3
chr10	96162547	96162548	G	A	TBC1D12	rs560424908	missense	1 of 13	Gag/Aag	E/K	60/775	49	0.340	0.001	15.25
chr4	164447360	164447361	T	G	MAR01.	rs76533100	3' UTR	6 of 6			-/289	32	0.222	0.058	15.25
chr5	14508346	14508347	A	T	TRIO	rs55900671	missense	57 of 57	aAg/aTg	K/M	3037/3097	60	0.417	0.058	15.17
chr17	8023903	8023904	C	A	ALOXE3	rs73241799	upstream				-/843	37	0.257	0.076	15.17
chr15	58246925	58246926	C	T	ALDH1A2	rs369448208	3' UTR	14 of 14			-/497	44	0.306	-1	15.16
chr17	53844646	53844647	C	T	PCTP	rs16956397	intronic				-/214	29	0.201	0.087	15.15
chr1	246890319	246890320	C	G	SCCPDH	rs78127097	intronic				-/429	29	0.201	0.097	15.14
chr19	38055552	38055553	T	C	ZNF571	rs16973890	missense	5 of 5	Aaa/Gaa	K/E	593/609	98	0.681	0.088	15.12
chr1	145116947	145116948	C	T	SEC22B	rs2794024	downstream					72	0.500	-1	15.11
chr1	145116452	145116453	C	G	SEC22B	rs111609380	non-coding exon	5 of 5				34	0.236	-1	15.06
chr17	77030414	77030415	G	T	C1QTNF1	rs77464894	5' UTR	2 of 5			-/281	31	0.215	0.075	15.06
chr3	62861041	62861042	T	G	CADPS	rs73844684	5' UTR	1 of 30			-/1353	29	0.201	0.089	15.04
chr17	21320710	21320711	C	G	KCNJ12	rs74883261	3' UTR	3 of 3			-/433	66	0.458	-1	15.03
chr3	97806304	97806305	G	C	OR5AC2	rs76749982	missense	1 of 1	Gct/Cct	A/P	97/309	49	0.340	0.047	14.99
chr12	12047473	12047474	T	C	ETV6	rs61921366	3' UTR	8 of 8			-/452	40	0.278	0.086	14.97
chr16	89007592	89007593	C	A	CBFA2T3	None	5' UTR	1 of 11			-/567	2	1.000	-1	14.95
chr1	111825357	111825358	G	A	CHIAP2	rs6662372	splice variant	4 of 9				29	0.201	0.030	14.95
chr6	136878579	136878580	G	T	MAP3K5	rs75456458	3' UTR	30 of 30			-/1374	36	0.250	0.076	14.95
chr1	86044074	86044075	T	C	CYR61	rs144472293	upstream				-/381	50	0.347	0.031	14.92
chr19	18174730	18174731	C	T	IL12RB1	rs11575935	missense	14 of 18	Gcg/Acg	A/T	525/662	59	0.410	0.053	14.89
chr1	19503935	19503936	A	T	UBR4	rs78463695	intronic				-/5183	32	0.222	0.063	14.88
chr5	121400271	121400272	A	G	LOX	rs79432032	3' UTR	7 of 7			-/417	46	0.329	0.042	14.83
chr1	228033257	228033258	T	A	PRSS38	rs61741607	missense	4 of 5	aTc/aAc	I/N	224/326	52	0.361	0.056	14.8
chr6	21596314	21596315	G	A	SOX4	rs79958549	3' UTR	1 of 1			-/474	64	0.444	0.035	14.79
chr17	2290849	2290850	T	G	MNT	rs140963967	missense	6 of 6	aAg/aCg	K/T	365/582	60	0.417	0.089	14.77
chr12	18793566	18793567	T	G	PIK3C2G	rs375680285	intronic				-/1445	38	0.271	-1	14.72
chr22	36136918	36136919	A	G	RBFOX2	rs550204644	3' UTR	12 of 12			-/367	50	0.347	0.012	14.67
chr11	105774532	105774533	T	C	GRIA4	rs61751525	splice variant				-/902	47	0.326	0.093	14.67
chr18	10671359	10671360	C	A	PIEZO2	rs376282306	3' UTR	14 of 14			-/709	52	0.361	-1	14.65
chr1	19231016	19231017	A	G	IFFO2	rs2295227	3' UTR	9 of 9			-/517	45	0.313	0.088	14.64
chr2	84932719	84932720	A	G	DNAH6	rs61750773	missense	51 of 76	aAg/aGg	K/R	2859/4158	38	0.264	0.068	14.62

chr2	86830875	86830876	C	A	RNF103	rs3755000	3' UTR	4 of 4			-/685	29	0.201	0.089	14.61
chr1	223307985	223307986	G	A	TLR5	rs45501199	intronic				-/858	50	0.347	0.090	14.61
chr3	45786342	45786343	T	C	SACM1L	rs11547070	3' UTR	20 of 20			-/587	72	0.500	-1	14.6
chr1	168215552	168215553	T	G	SFT2D2	rs3795609	3' UTR	8 of 8			-/160	77	0.535	0.087	14.59
chr1	173505121	173505122	A	G	SLC9C2	None	intronic				-/450	29	0.204	-1	14.57
chr8	39607104	39607105	C	T	ADAM2	rs77172402	intronic				-/716	27	0.471	-1	14.56
chr3	124946369	124946370	G	A	ZNF148	rs2292520	3' UTR	9 of 9			-/794	30	0.208	0.029	14.54
chr8	146223804	146223805	A	G	ZNF252P	rs115186140	intronic					48	0.333	0.061	14.54
chr1	225237949	225237950	A	G	DNAH14	rs78320839	missense	15 of 83	Atc/Gtc	I/V	651/4515	49	0.340	0.018	14.53
chr1	204161946	204161947	C	T	KISS1	rs12998	missense	2 of 3	Gag/Aag	E/K	20/138	29	0.201	0.058	14.53
chr2	68479733	68479734	C	G	PPP3R1	None	intronic				-/189	14	0.875	-1	14.51
chr3	182810143	182810144	T	G	MCCC1	rs3732604	intronic				-/616	80	0.556	-1	14.5
chr13	101757010	101757011	A	C	NALCN	rs16958350	intronic				-/1738	48	0.333	0.070	14.49
chr8	87588042	87588043	C	T	CNGB3	rs375288585	missense	18 of 18	Gct/Act	A/T	807/809	48	0.333	0	14.48
chr9	123205980	123205981	C	T	CDK5RAP2	rs34523498	missense	8 of 23	gGa/gAa	G/E	416/1287	51	0.354	0.041	14.48
chr1	1686080	1686081	G	A	NADK	rs75816936	missense	6 of 10	Cgg/Tgg	R/W	217/414	68	0.472	0.055	14.48
chr8	105360993	105360994	C	A	DCSTAMP	rs61682032	missense	1 of 2	Ctg/Atg	L/M	72/283	34	0.236	0.079	14.48
chr1	186107190	186107191	T	C	HMCN1	rs117650377	intronic				-/5635	37	0.257	0.023	14.47
chr22	30661027	30661028	T	C	OSM	rs148386625	missense	2 of 3	gAt/gGt	D/G	47/252	116	0.806	0.006	14.46
chr3	142682072	142682073	C	T	PAQR9	rs115958571	missense	1 of 1	Gac/Aac	D/N	36/377	37	0.257	0.089	14.46
chr8	92082752	92082753	C	T	OTUD6B	rs3808378	intronic				-/192	124	0.861	-1	14.37
chr8	87573073	87573074	T	C	CPNE3	rs369591662	3' UTR	16 of 16			-/537	44	0.306	-1	14.36
chr2	165350910	165350911	T	C	GRB14	rs115094739	intronic				-/453	47	0.326	0.037	14.34
chr10	20534255	20534256	G	A	PLXDC2	rs774005859	intronic				-/529	32	0.222	0	14.29
chr17	3626552	3626553	C	A	ITGAE	rs62072592	intronic				-/1179	44	0.306	0	14.28
chr1	225519115	225519116	A	G	DNAH14	rs41304141	intronic				-/4515	51	0.354	0.072	14.28
chr3	4793369	4793370	C	T	ITPR1	rs761700426	intronic				-/2743	32	0.222	-1	14.26
chr2	190620215	190620216	C	T	OSGEPL1	rs75321854	missense	3 of 4	Gct/Act	A/T	98/222	50	0.347	0.070	14.26
chr2	68479722	68479723	G	A	PPP3R1	rs184336391	intronic				-/189	64	0.744	-1	14.24
chr17	21323073	21323074	T	C	KCNJ12	rs73981249	3' UTR	3 of 3			-/433	32	0.250	-1	14.2
chr3	56650011	56650012	G	A	CCDC66	rs4681904	missense	12 of 13	Gaa/Aaa	E/K	548/633	1	0.266	0.071	14.19
chr2	160599716	160599717	C	G	MAR07.	rs17813964	missense	4 of 4	aCt/aGt	T/S	100/101	33	0.229	0.090	14.19
chr4	130023883	130023884	G	T	C4orf33	rs35199409	missense	2 of 4	aGg/aTg	R/M	40/102	59	0.410	0.094	14.16

chr14	96553065	96553066	C	T	C14orf132	rs537320987	5' UTR	2 of 2			-/173	51	0.354	0.002	14.15
chr5	176722004	176722005	G	A	NSD1	rs78247455	missense	23 of 23	Gcc/Acc	A/T	2546/2696	33	0.229	0.058	14.15
chr1	175046825	175046826	G	A	TNN	rs41266078	missense	2 of 19	cGc/cAc	R/H	91/1299	32	0.222	0.052	14.14
chr3	12538110	12538111	A	G	TSEN2	rs41293387	intronic				-/465	35	0.243	0.078	14.13
chr8	102701455	102701456	C	A	NCALD	rs200190236	3' UTR	7 of 7			-/193	18	0.396	-1	14.11
chr12	111082835	111082836	G	T	TCTN1	rs118096349	missense	12 of 15	Ggc/Tgc	G/C	452/573	35	0.243	0.075	14.11
chr6	157710748	157710749	C	G	TMEM242	None	3' UTR	4 of 4			-/141	53	0.368	-1	14.09
chr19	58118370	58118371	G	A	ZNF530	rs78803667	missense	3 of 3	tGc/tAc	C/Y	493/599	39	0.271	0.056	14.08
chr9	95063946	95063947	C	T	NOL8	rs921122	missense	14 of 18	Ggt/Agt	G/S	953/1099	34	0.236	0.093	14.06
chr3	130308037	130308038	T	A	COL6A6	rs77685098	intronic				-/2263	29	0.201	0.079	14.04
chr16	28503035	28503036	C	T	CLN3	rs201824641	splice variant	3 of 5	gaG/gaA	E	15/74	59	0.410	0.025	14.03
chr18	29867687	29867688	T	C	GAREM	rs3744921	missense	4 of 6	aAg/aGg	K/R	291/875	33	0.229	0.055	14.03
chr2	180036774	180036775	G	A	SESTD1	rs10497534	intronic				-/143	28	0.209	0.085	14.01
chr7	27239810	27239811	G	T	HOXA13	None	upstream				-/388	2	0.500	-1	14
chr7	156474168	156474169	T	C	LMBR1	rs1014236	3' UTR	17 of 17			-/490	55	0.382	0.094	13.96
chr15	63111738	63111739	T	G	TLN2	None	missense	50 of 56	Ttc/Gtc	F/V	2266/2542	3	0.330	0	13.95
chr18	47349775	47349776	A	G	MYO5B	rs78201339	3' UTR	40 of 40			-/1848	30	0.208	-1	13.94
chr8	23294549	23294550	T	C	ENTPD4	rs371948028	missense	10 of 13	cAg/cGg	Q/R	424/616	35	0.243	0.003	13.93
chr20	50384949	50384950	C	A	ATP9A	None	5' UTR	1 of 28			-/1047	1	0.250	-1	13.92
chr6	139568909	139568910	A	G	TXLNB	rs373639261	intronic				-/684	32	0.222	0	13.92
chr5	159840934	159840935	T	A	SLU7	rs41275313	missense	4 of 16	Atg/Ttg	M/L	126/586	50	0.347	0.077	13.91
chr8	22277702	22277703	T	C	SLC39A14	rs76963096	3' UTR	9 of 9			-/492	67	0.465	0.094	13.89
chr15	21934162	21934163	C	A	RP11-32B5.1	rs12438448	downstream					43	0.299	-1	13.85
chr14	39901794	39901795	C	A	FBXO33	None	upstream				-/555	2	0.500	-1	13.84
chr11	62910890	62910891	C	A	SLC22A24	rs116409312	missense	1 of 4	Gtg/Ttg	V/L	121/322	31	0.215	0.028	13.84
chr2	163374140	163374141	C	T	KCNH7	rs368505693	intronic				-/1196	30	0.208	0.007	13.83
chr3	194309148	194309149	A	T	TMEM44	rs73195025	3' UTR	11 of 11			-/439	33	0.229	0.055	13.82
chr13	26789637	26789638	T	C	RNF6	rs12428716	missense	5 of 6	gAc/gGc	D/G	6/329	49	0.340	0.098	13.81
chr17	21280223	21280224	A	C	KCNJ12	None	splice variant	1 of 3			-/433	2	0.500	-1	13.8
chr13	52960408	52960409	C	T	THSD1	rs61958012	intronic				-/473	52	0.361	0.065	13.79
chr6	139498627	139498628	A	G	HECA	rs3777670	3' UTR	4 of 4			-/543	29	0.201	0.060	13.77
chr1	42776698	42776699	G	A	FOXJ3	rs200354449	intronic				-/622	6	0.375	0.004	13.76
chr2	179411010	179411011	T	C	TTN	rs72648257	missense	170 of 191	Agt/Ggt	S/G	22618/26926	54	0.375	0.065	13.76

chr7	107427321	107427322	A	C	SLC26A3	rs34407351	missense	8 of 21	tgT/tgG	C/W	307/764	35	0.243	0.067	13.76
chr16	66586432	66586433	C	A	TK2	None	5' UTR	1 of 8			-/88	33	0.229	-1	13.75
chr12	6862291	6862292	A	G	MLF2	rs117966544	5' UTR	1 of 9			-/248	76	0.528	0.087	13.75
chr14	39901795	39901796	G	T	FBXO33	None	upstream				-/89	2	0.500	-1	13.74
chr10	45499271	45499272	C	G	ZNF22	rs3740092	synonymous	2 of 2	tcC/tcG	S	152/224	37	0.257	0.089	13.71
chr19	50879768	50879769	T	C	NR1H2	rs73932482	5' UTR	1 of 6			-/209	47	0.326	0.061	13.7
chr14	23352397	23352398	T	G	REM2	rs868062878	5' UTR	1 of 5			-/340	25	0.202	-1	13.66
chr16	75661553	75661554	C	T	ADAT1	rs373571902	upstream				-/502	28	0.212	-1	13.65
chr3	32280150	32280151	C	A	CMTM8	rs62243275	upstream				-/115	64	1.000	-1	13.63
chr17	21320698	21320699	G	T	KCNJ12	rs72842113	3' UTR	3 of 3			-/433	72	0.500	-1	13.63
chr15	68120304	68120305	C	A	SKOR1	rs114784960	missense	2 of 9	gaC/gaA	D/E	674/926	34	0.283	0.059	13.62
chr12	78225298	78225299	G	A	NAV3	rs61754235	missense	1 of 40	Gtg/Atg	V/M	20/2385	51	0.354	0.073	13.62
chr22	50354414	50354415	G	A	PIM3	None	5' UTR	1 of 6			-/326	2	0.200	-1	13.61
chr2	70902822	70902823	C	T	ADD2	rs116545672	3' UTR	13 of 13			-/559	70	0.486	0.095	13.61
chr16	73126496	73126497	G	A	HCCAT5	rs368807617	non-coding exon	1 of 3				35	0.243	-1	13.59
chr19	41895838	41895839	C	T	EXOSC5	None	intronic				-/62	8	0.479	0.025	13.59
chr17	21280218	21280219	G	T	KCNJ12	None	5' UTR	1 of 3			-/433	2	0.500	-1	13.57
chr17	21311767	21311768	G	C	KCNJ12	rs55744777	intronic				-/433	72	0.500	-1	13.57
chr17	21323106	21323107	A	G	KCNJ12	rs73981251	3' UTR	3 of 3			-/433	23	0.213	-1	13.55
chr6	395798	395799	A	G	IRF4	rs374609552	intronic				-/451	50	0.347	0.001	13.55
chr3	197476391	197476392	C	G	KIAA0226	rs116250860	5' UTR	1 of 21			-/927	73	0.507	0.068	13.54
chr17	35913170	35913171	T	C	SYNRG	rs78515247	intronic				-/1224	33	0.229	0.028	13.53
chr6	44145154	44145155	G	T	CAPN11	rs147936977	missense	12 of 23	Gcg/Tcg	A/S	472/739	29	0.201	0.005	13.48
chr6	46851295	46851296	C	T	GPR116	rs78333594	splice variant	6 of 21	gcG/gcA	A	204/1346	57	0.396	0.080	13.47
chr15	40861964	40861965	G	C	RPUSD2	rs34974852	splice variant				-/484	42	0.292	0.033	13.46
chr1	144851553	144851554	T	A	PDE4DIP	rs3736	3' UTR	44 of 44			-/2362	46	0.319	-1	13.44
chr9	100778040	100778041	C	T	ANP32B	rs200316910	3' UTR	7 of 7			-/251	48	0.333	-1	13.44
chr17	21280222	21280223	G	T	KCNJ12	None	splice variant	1 of 3			-/433	2	0.500	-1	13.44
chr22	50659698	50659699	G	A	TUBGCP6	rs751897821	missense	16 of 25	tCa/tTa	S/L	1030/1819	41	0.285	0	13.43
chr1	145116832	145116833	C	T	SEC22B	rs5015527	non-coding exon	5 of 5				72	0.500	-1	13.41
chr16	16236788	16236789	A	G	ABCC1	rs148591472	3' UTR	30 of 30			-/1472	35	0.243	0.043	13.4
chr10	96234440	96234441	C	T	TBC1D12	rs377630453	missense	3 of 13	aCg/aTg	T/M	371/775	49	0.340	0	13.38
chr15	48583929	48583930	T	C	SLC12A1	rs35718278	intronic				-/1099	33	0.229	0.037	13.38

chrX	152936392	152936393	T	A	PNCK	rs56060609	missense	9 of 12	caA/caT	Q/H	262/343	103	0.715	0	13.37
chr20	826207	826208	T	C	FAM110A	rs191347965	missense	1 of 2	gTg/gCg	V/A	148/189	36	0.250	0.022	13.37
chr13	103696389	103696390	T	C	SLC10A2	None	3' UTR	6 of 6			-/348	29	0.201	-1	13.36
chr3	42660488	42660489	C	T	NKTR	None	intronic				-/1462	10	0.469	-1	13.34
chr16	71889172	71889173	C	G	ATXN1L	rs6499545	3' UTR	3 of 3			-/689	1	0.500	0.005	13.34
chr19	51645996	51645997	G	T	SIGLEC7	rs368230574	missense	1 of 2	cGt/cTt	R/L	124/145	38	0.264	0.009	13.34
chr19	51833626	51833627	A	G	VSIG10L	rs150438071	downstream				-/867	39	0.271	0.011	13.34
chr11	8124451	8124452	T	C	TUB	rs17847551	3' UTR	13 of 13			-/561	37	0.325	0.088	13.32
chr4	4420336	4420337	A	G	NSG1	rs7673866	3' UTR	8 of 8			-/185	38	0.264	0.095	13.32
chr6	132910745	132910746	G	A	TAAR5	rs369553631	missense	1 of 17	aCa/aTa	T/I	27/337	36	0.250	0.001	13.31
chr16	57764894	57764895	C	T	CCDC135	rs115337501	missense	17 of 18	aCg/aTg	T/M	815/874	52	0.361	0.039	13.31
chr6	135260502	135260503	T	G	ALDH8A1	rs777242234	missense	4 of 6	Ata/Cta	I/L	165/433	48	0.333	0	13.29
chr3	16306574	16306575	G	A	OXNAD1	rs111654033	upstream				-/330	34	0.236	0.031	13.27
chr15	21938048	21938049	C	T	RP11-32B5.7	rs2343906	downstream					101	0.701	-1	13.22
chr9	19408985	19408986	C	T	ACER2	rs377429359	5' UTR	1 of 5			-/140	45	0.313	-1	13.2
chr22	50354415	50354416	G	A	PIM3	None	5' UTR	1 of 6			-/326	2	0.200	-1	13.2
chr12	50026422	50026423	C	T	PRPF40B	rs79252534	intronic				-/858	30	0.208	0.067	13.19
chr8	143846039	143846040	G	A	LYNX1	rs371168001	missense	5 of 5	Ctc/Ttc	L/F	127/131	62	0.431	0	13.18
chr22	32587285	32587286	C	T	RFPL2	rs142520857	missense	5 of 5	Gac/Aac	D/N	204/378	60	0.417	0.076	13.17
chr5	35230379	35230380	G	C	PRLR	rs10068521	5' UTR	1 of 5			-/34	33	0.229	0.086	13.17
chr17	80478088	80478089	G	T	FOXK2	None	missense	1 of 9	Ggc/Tgc	G/C	109/660	2	0.250	-1	13.15
chr1	39758438	39758439	G	T	MACF1	rs139995582	missense	17 of 101	gGa/gTa	G/V	639/7555	53	0.368	0.014	13.13
chr9	127713621	127713622	A	G	SCAI	None	3' UTR	18 of 18			-/606	2	0.200	-1	13.1
chr9	98279233	98279234	C	T	PTCH1	rs118133906	5' UTR	1 of 5			-/183	30	0.208	0.022	13.08
chr17	74469778	74469779	T	C	RHBDF2	rs73998915	missense	15 of 19	Atg/Gtg	M/V	562/827	49	0.340	0.036	13.08
chr10	72300884	72300885	C	T	PALD1	rs117803924	missense	16 of 20	Cgg/Tgg	R/W	646/856	53	0.368	0.039	13.08
chr17	8110939	8110940	G	A	AURKB	rs55878091	missense	3 of 8	gCc/gTc	A/V	52/312	73	0.507	0.036	13.06
chr14	96829586	96829587	G	A	ATG2B	rs180722527	5' UTR	1 of 42			-/2078	37	0.257	0.076	13.06
chr19	5690303	5690304	G	A	RPL36	rs3810220	5' UTR	3 of 6			-/105	37	0.257	0.027	13.01
chr17	40274890	40274891	T	C	HSPB9	rs73311647	missense	1 of 1	tTc/tCc	F/S	8/159	42	0.292	0.061	13.01
chr3	191860310	191860311	T	C	FGF12	None	3' UTR	5 of 5			-/181	38	0.264	-1	12.99
chr3	194076005	194076006	G	A	LRRC15	rs6578	3' UTR	2 of 2			-/587	34	0.236	0.096	12.99
chr17	8191937	8191938	C	T	RANGRF	rs111476121	5' UTR	1 of 5			-/186	93	0.646	0.072	12.97

Table S3. Results of gene set enrichment analysis in Enrichr* using 72 genes with 81 variants low burden variants classified as pathogenic in ClinVar. Only results where the Z score is <-1 or >1, and the enrichment adjusted P-value is <0.01, are included. Enrichr contains a collection of diverse gene set libraries. Here we provide results for enrichment of genes in our dataset relevant to cardiovascular disease, renal disease, or diabetes, compared to the Reactome 2016, Wiki 2016 and KEGG 2016 pathways databases, the 2017gene ontology (GO) databases for biological processes and cellular components, and the OMIM Diseases Table.

Database	Term	P-value	Adjusted P-value	Z-score	Combined Score	Genes
Reactome 2016	Metabolism_Homo sapiens_R-HSA-1430728	4.75E-09	1.248E-06	-2.25	43.04	GCDH;GALT;FECH;LPL;SLC4A1;DHTKD1;ALAD;APOE; PRODH;UGT1A8;ACADS;**ARSA**;NQO1;ABCG8;NOS3; GAA;APOC3;AMPD1;APOA4;FMO3;TYR;GALC; GNPAT; BTD;RHAG
	Chylomicron-mediated lipid transport_Homo sapiens_R-HSA-174800	3.55E-07	4.66E-05	-1.88	27.90	LPL;APOC3;APOA4;APOE
	Transmembrane transport of small molecules_Homo sapiens_R-HSA-382551	8.45E-06	4.45E-04	-2.10	24.52	GLRA1;ABCG8;SLC22A18;SCNN1A;ABCC6;RHAG; ATP6V0A4;SLC4A1;SLC6A20;CFTR;FTL
	Lipoprotein metabolism_Homo sapiens_R-HSA-174824	6.60E-06	4.34E-04	-2.02	24.07	APOC3;LPL;APOA4;APOE
	Lipid digestion, mobilization, and transport_Homo sapiens_R-HSA-73923	5.68E-06	4.34E-04	-1.81	21.85	ABCG8;APOC3;LPL;APOA4;APOE
	Retinoid metabolism and transport_Homo sapiens_R-HSA-975634	1.56E-05	6.83E-04	-1.91	21.12	APOC3;LPL;APOA4;APOE
Wiki Pathways 2016	Statin Pathway_Homo sapiens_WP430	8.29E-08	1.05E-05	-1.81	29.58	ABCG8;APOC3;LPL;APOA4;APOE
	Regulation of Cardiac Hypertrophy by miR-208_Mus musculus_WP1526	8.88E-07	5.59E-05	-1.03	14.37	MSTN;GJA5;GATA4
	Composition of Lipid Particles_Homo sapiens_WP3601	3.70E-06	1.55E-04	-1.53	19.19	APOC3;LPL;APOE
	Statin Pathway_Mus musculus_WP1	2.93E-05	9.24E-04	-1.60	16.69	LPL;APOA4;APOE
KEGG 2016	Metabolic pathways_Homo sapiens_hsa01100	1.37E-06	1.43E-04	-2.01	27.15	GCDH;GALT;ALG6;FECH;NOS3;GAA;AMPD1;TYR; ALAD;GALC;C1GALT1C1;BTD;ATP6V0A4;PIGL; PRODH;ACADS;UGT1A8
	Lysosome_Homo sapiens_hsa04142	1.01E-03	0.026	-1.70	11.71	GALC;**ARSA**;GAA;ATP6V0A4
GO Biological Processes 2017	Chylomicron remodeling (GO:0034371)	1.92E-08	2.88E-05	-2.75	48.87	LPL;APOC3;APOA4;APOE
	Cholesterol homeostasis (GO:0042632)	2.47E-08	2.88E-05	-2.80	49.04	ABCG8;CAV3;APOC3;LPL;APOA4;APOE
	Triglyceride metabolic process (GO:0006641)	8.82E-07	6.06E-04	-2.65	36.95	CAV3;LPL;APOC3;APOE
	Triglyceride homeostasis (GO:0070328)	1.08E-06	6.06E-04	-3.03	41.70	LPL;APOC3;APOA4;APOE
	Very-low-density lipoprotein particle remodeling (GO:0034372)	2.47E-06	8.64E-04	-2.30	29.74	LPL;APOA4;APOE
	Chylomicron assembly (GO:0034378)	5.27E-06	1.18E-03	-3.01	36.56	APOC3;APOA4;APOE
	Cholesterol efflux (GO:0033344)	5.84E-06	1.18E-03	-2.57	30.98	ABCG8;APOC3;APOA4;APOE
	High-density lipoprotein particle remodeling (GO:0034375)	2.93E-05	3.80E-03	-2.53	26.40	APOC3;APOA4;APOE
	Triglyceride catabolic process (GO:0019433)	8.55E-05	6.24E-03	-2.38	22.34	CAV3;APOC3;LPL;APOE
	Removal of superoxide radicals (GO:0019430)	9.72E-05	0.008	-2.53	23.41	NOS3;APOA4;SOD3
	Ventricular cardiac muscle cell action potential (GO:0086005)	9.72E-05	8.40E-03	-3.33	30.74	KCNE1;CAV3;SCN5A
GO Cellular Component 2017	endoplasmic reticulum lumen (GO:0005788)	4.8E-06	4.93E-04	-2.87	35.17	CST3;**ARSA**;COL18A1;SERPINA1;COL6A3;GHRL; APOA4;APOE
	Very-low-density lipoprotein particle (GO:0034361)	1.97E-05	9.70E-04	-1.36	14.75	APOC3;APOA4;APOE
	early endosome (GO:0005769)	2.07E-04	2.61E-03	-2.37	20.13	HFE;APOC3;APOA4;APOE;CFTR
	lysosomal membrane (GO:0005765	2.49E-04	2.61E-03	-3.03	25.15	GALC;**ARSA**;KCNE1;GAA;ATP6V0A4;TYR;CFTR
	lysosome (GO:0005764)	3.50E-04	2.61E-03	-2.30	18.30	GALC;**ARSA**;KCNE1;GAA;TYR
OMIM Disease Table	Long_qt_syndrome	9.62E-06	1.88E-04	-1.07	12.41	KCNE1;CAV3;SCN5A
	Cardiomyopathy	1.56E-05	1.88E-04	-1.86	20.60	CAV3;LDB3;SCN5A;TTN
	Myopathy	1.88E-05	1.88E-04	-1.46	15.85	CAV3;COL6A3;LDB3;TTN
	Cardiomyopathy, hypertrophic	1.70E-03	5.60E-03	1.84	-11.76	CAV3;TTN

* Chen, E.Y. et al. Enrichr: interactive and collaborative HTML5 gene list enrichment analysis tool. BMC Bioinformatics 14, 128 (2013).
Kuleshov, M.V. et al. Enrichr: a comprehensive gene set enrichment analysis web server 2016 update. Nucleic Acids Res 44, W90-7 (2016)

Table S4. Haplotype analysis for association between T2D and sliding windows of 2, 3, 4, 5 and 6 SNP haplotypes across the ARSA gene. Logistic regression analysis under an additive model with 10 PCs as covariates.

Gene		ARSA					
Variant		rs11912237	rs8142033	rs6151429	rs2071421	rs6151419	rs762668
Haplotype	Frequency	Risk Haplotypes					
AA	0.43	OR 2.67; P = 2.43×10^{-4}					
AG	0.43		OR 2.67; P = 2.43×10^{-4}				
GG	0.43			OR 2.67; P = 2.43×10^{-4}			
GA	0.43				OR 2.91; P = 7.61×10^{-5}		
AA	0.41					OR 2.67; P = 3.21×10^{-4}	
Haplotype	Frequency	Protective Haplotypes					
GG	0.57	OR 0.37; P = 2.43×10^{-4}					
GA	0.57		OR 0.37; P = 2.43×10^{-4}				
AA	0.57			OR 0.37; P = 1.54×10^{-4}			
AG	0.51				OR 0.37; P = 0.003		
GG	0.50					OR 0.47; P = 0.003	

Gene		ARSA					
Variant		rs11912237	rs8142033	rs6151429	rs2071421	rs6151419	rs762668
Haplotype	Frequency	Risk Haplotypes					
AAG	0.43	OR 2.67; P = 2.43×10^{-4}					
AGG	0.43		OR 2.67; P = 2.43×10^{-4}				
GGA	0.42			OR 2.85; P = 1.21×10^{-4}			
GAA	0.41				OR 2.67; P = 3.21×10^{-4}		
Haplotype	Frequency	Protective Haplotypes					
GGA	0.57	OR 0.37; P = 2.43×10^{-4}					
GAA	0.57		OR 0.37; P = 1.54×10^{-4}				
AAG	0.51			OR 0.47; P = 0.003			
AGG	0.50				OR 0.47; P = 0.003		

Gene		ARSA					
Variant		rs11912237	rs8142033	rs6151429	rs2071421	rs6151419	rs762668
Haplotype	Frequency	Risk Haplotypes					
AAGG	0.43	OR 2.67; P = 2.43×10^{-4}					
AGGA	0.42		OR 2.85; P = 1.21×10^{-4}				
GGAA	0.41			OR 2.67; P = 3.21×10^{-4}			
Haplotype	Frequency	Protective Haplotypes					
GGAA	0.57	OR 0.37; P = 1.54×10^{-4}					
GAAG	0.51		OR 0.47; P = 0.003				
AAGG	0.50			OR 0.47; P = 0.003			

Gene		ARSA					
Variant		rs11912237	rs8142033	rs6151429	rs2071421	rs6151419	rs762668
Haplotype	Frequency	Risk Haplotypes					
AAGGA	0.42	OR 2.85; P = 1.21×10^{-4}					
AGGAA	0.41		OR 2.67; P = 3.21×10^{-4}				
Haplotype	Frequency	Protective Haplotypes					
GGAAG	0.51	OR 0.47; P = 0.003					
GAAGG	0.50		OR 0.47; P = 0.003				

Gene		ARSA					
Variant		rs11912237	rs8142033	rs6151429	rs2071421	rs6151419	rs762668
Haplotype	Haplotype	Risk Haplotypes					
AAGGAA	0.41	OR 2.67; P = 3.21×10^{-4}					
Haplotype	Frequency	Protective Haplotypes					
GGAAGG	0.50	OR 0.47; P = 0.003					

Table S5. Characteristics of subjects for WES and GWAS analyses.

Clinical Variable	WES Adults (N=48)	WES Adults eGFR<60 (N=21)	WES Adults eGFR>60 (N=27)	WES Minors (N=22)	GWAS All (N=391)	GWAS T2D (N=73)
Age (years)	53±16	64±12	44±13	13±8	25±19	47±14
Gender (M:F)	19:29	14:07	05:22	14:10	179:212	22:51
DBP (mm Hg)	98.9±13.3	104.3±10.9	95.2±13.7	NA	88.1±17.9	102.3±10.7
SBP (mm Hg)	161.5±25.6	179.2±22.5	148.7±19.6	NA	140.9±27.3	166.8±21.6
ACR	143±242	252±326	65±111	NA	65±159	137±210
eGFR	70.6±39.7	34.0±14.8	99.0±27.5	NA	95.5±37.4	83.3±34.2
HbA1c	9.0±3.5	8.8±4.2	9.2±2.9	NA	8.4±3.2	11.0±2.7
BMI	36.6±13.3	30.1±10.6	41.7±13.5	NA	26.0±10.0	35.6±7.7

Notes: N values vary according to trait, samples sizes by trait and phenotype/ trait are included on all graphs. The N=48 WES adults were selected on the basis of eGFR indicative of moderate (eGFR30-59; N=10)/severe (eGFR15-28; N=10))/kidney failure (eGFR<15; N=1) CRD versus normal kidney function (eGFR>60; N=27). Values are Mean±SD.

Figure S1.

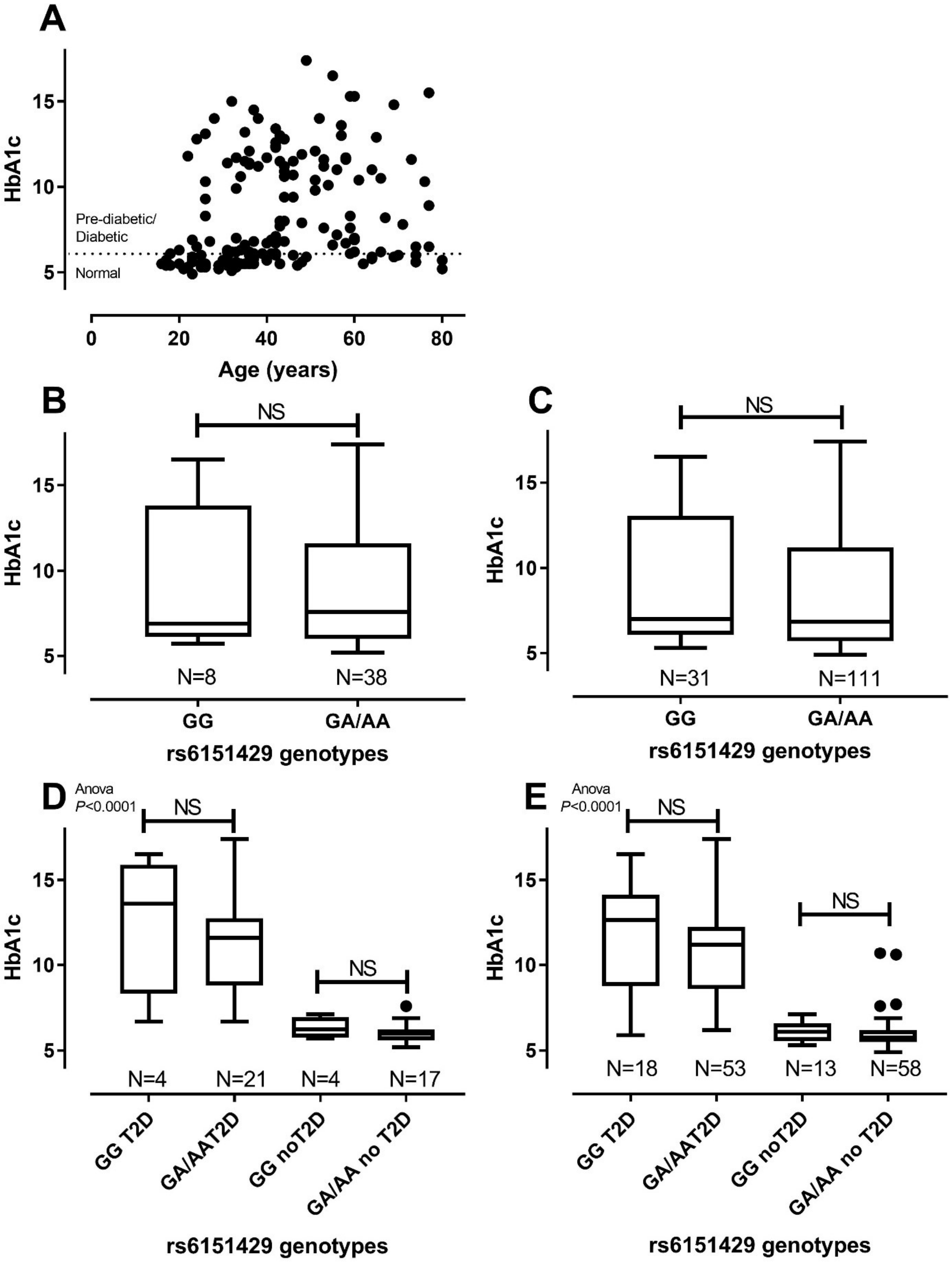

Figure S1. Relationship between ARSA rs6151429 genotypes and HbA1c in the study population. (A) shows HbA1c by age for all individuals contributing to the GWAS*; the dotted line indicates the clinical cut-off for pre-T2D and T2D. (B) and (C) show Box and Whiskers Tukey plots for genotype by HbA1c for WES and GWAS participants, respectively. (D) and (E) show results for WES and GWAS participants, respectively, stratified by T2D status. *Anderson et al., 2015, *PLOS ONE*, 10(3):e0119333.

Figure S2.

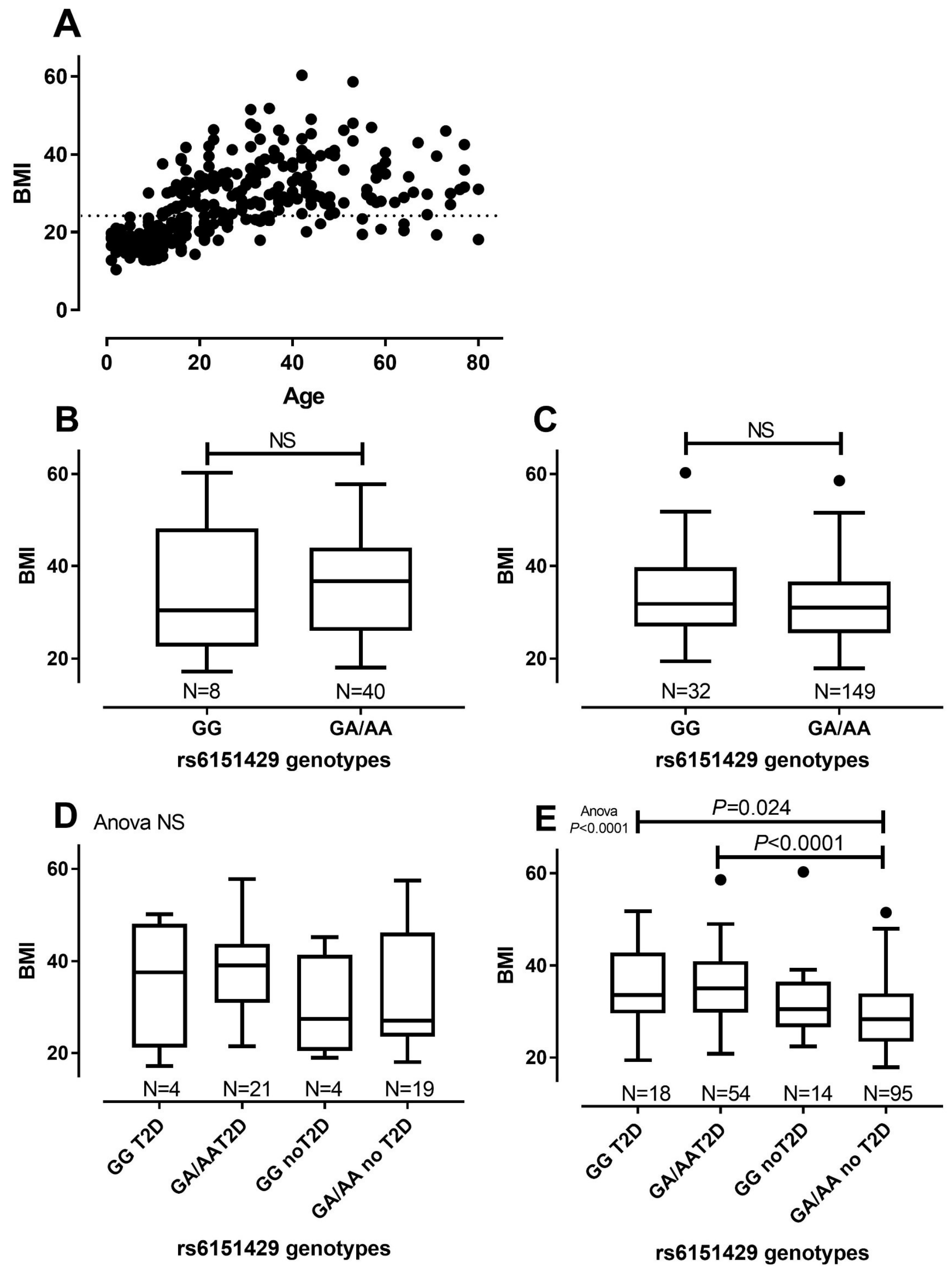

Figure S2. Relationship between ARSA rs6151429 genotypes and BMI in the study population. (A) shows BMI by age for all individuals contributing to the GWAS*; the dotted line indicates the clinical cut-off for T2D risk. (B) and (C) show Box and Whiskers Tukey plots for genotype by BMI for WES and GWAS participants, respectively. (D) and (E) show results for WES and GWAS participants, respectively, stratified by T2D status. *Anderson et al., 2015, *PLOS ONE*, 10(3):e0119333.

Figure S3.

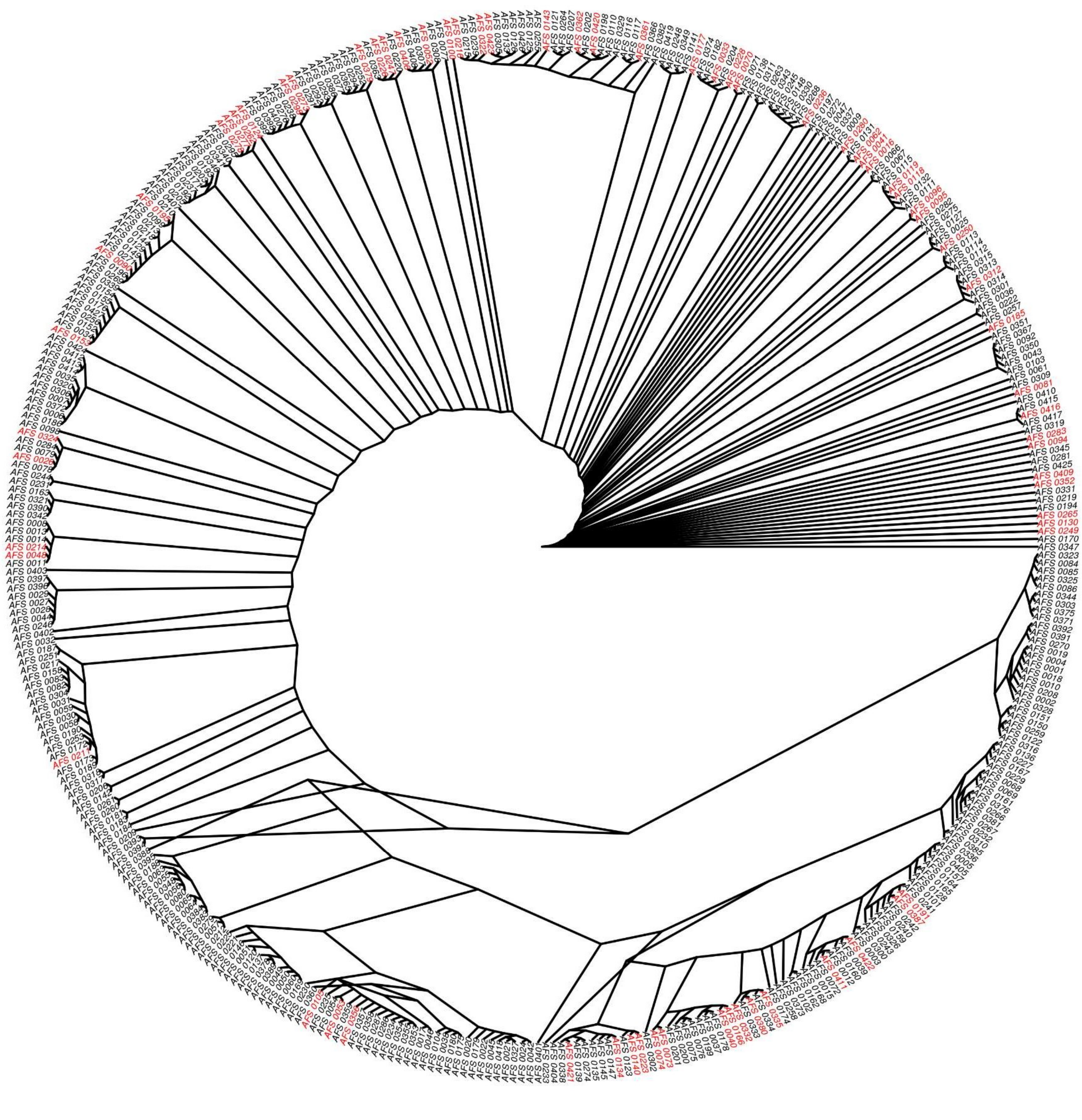

Figure S3. Radial plot showing hierarchical clustering of estimated pairwise identity-by-descent allele-sharing for the 402 genotyped individuals used in the original GWAS*. The genomic kinship matrix was first calculated in GenABEL v1.7-6, and converted to a distance matrix and hierarchical cluster analysis using single linkage on the dissimilarities. The ape package was used to produce radial tree plot. The individuals for whom WES was carried out in this study are highlighted in red. Adapted from Figure S1 of *Anderson et al., 2015, *PLOS ONE*, 10(3):e0119333.
